# Global biogeography of chemosynthetic symbionts reveals both localized and globally distributed symbiont groups

**DOI:** 10.1073/pnas.2104378118

**Published:** 2021-07-16

**Authors:** Jay T. Osvatic, Laetitia G. E. Wilkins, Lukas Leibrecht, Matthieu Leray, Sarah Zauner, Julia Polzin, Yolanda Camacho, Olivier Gros, Jan A. van Gils, Jonathan A. Eisen, Jillian M. Petersen, Benedict Yuen

**Affiliations:** ^a^Centre for Microbiology and Environmental Systems Science, University of Vienna, 1090 Vienna, Austria;; ^b^Genome and Biomedical Sciences Facility, Genome Center, University of California, Davis, CA 95616;; ^c^Department of Symbiosis, Max Planck Institute for Marine Microbiology, 28209 Bremen, Germany;; ^d^Smithsonian Tropical Research Institute, Apartado 0843–03092, Balboa, Republic of Panama;; ^e^Centro de Investigación en Ciencias del Mar y Limnología, Escuela de Biología, Universidad de Costa Rica, San Pedro 11501-2060, Costa Rica;; ^f^UMR 7205, Institut de Systématique, Évolution, Biodiversité, Equipe Biologie de la Mangrove, Département de Biologie, Université des Antilles, 97159 Pointe-à-Pitre Cedex, Guadeloupe;; ^g^Royal Netherlands Institute for Sea Research,1790 AB Den Burg, The Netherlands;; ^h^Department of Evolution and Ecology, University of California, Davis, CA 95616;; ^i^Department of Medical Microbiology and Immunology, University of California, Davis, CA 95616

**Keywords:** symbiosis, biogeography, recombination

## Abstract

In the ocean, most hosts acquire their symbionts from the environment. Due to the immense spatial scales involved, our understanding of the biogeography of hosts and symbionts in marine systems is patchy, although this knowledge is essential for understanding fundamental aspects of symbiosis such as host–symbiont specificity and evolution. Lucinidae is the most species-rich and widely distributed family of marine bivalves hosting autotrophic bacterial endosymbionts. Previous molecular surveys identified location-specific symbiont types that “promiscuously” form associations with multiple divergent cooccurring host species. This flexibility of host–microbe pairings is thought to underpin their global success, as it allows hosts to form associations with locally adapted symbionts. We used metagenomics to investigate the biodiversity, functional variability, and genetic exchange among the endosymbionts of 12 lucinid host species from across the globe. We report a cosmopolitan symbiont species, *Candidatus* Thiodiazotropha taylori, associated with multiple lucinid host species. *Ca.* T. taylori has achieved more success at dispersal and establishing symbioses with lucinids than any other symbiont described thus far. This discovery challenges our understanding of symbiont dispersal and location-specific colonization and suggests both symbiont and host flexibility underpin the ecological and evolutionary success of the lucinid symbiosis.

## Significance

Knowledge of host–symbiont biogeography is critical to understanding fundamental aspects of symbiosis such as host–symbiont specificity. Marine animals typically acquire their symbionts from the environment, a strategy that enables the host to associate with symbionts that are well-suited to local conditions. In contrast, we discovered that in the chemosymbiotic bivalve family Lucinidae several host species distributed across the globe are all associated with a single cosmopolitan bacterial symbiont. The genetic cohesiveness of this global symbiont species is maintained through homologous recombination across its extensive geographical range. The remarkable flexibility in the lucinid association is advantageous to both host and symbiont as it increases the likelihood of locating a compatible partner across diverse habitats spanning the globe.

Nutritional symbioses between eukaryotic organisms and autotrophic microbes are ubiquitous throughout Earth’s oceans. These associations have allowed marine organisms to flourish in nutrient-limited or extreme environments where they reach population densities unmatched by their nonsymbiotic relatives (1, 2). Having lost crucial biosynthesis pathways or the entire digestive tract, hosts in autotrophic nutritional symbioses are obligatorily dependent on the photosynthetic or chemosynthetic metabolisms of their symbionts for survival (3, 4). However, many photosymbiotic and chemosymbiotic hosts do not vertically transmit their symbionts and each new generation must acquire their symbionts from the environment (5). One potential benefit of this strategy is the opportunity to partner with symbionts better suited to the local conditions in which a larva settles and develops. Horizontal transmission thus creates the opportunity for hosts to associate with a greater variety of symbionts, and the degree of flexibility in obligate nutritional symbioses has been subject to much research (6–10).

Members of the bivalve family Lucinidae form an obligate association with chemolithoautotrophic gammaproteobacteria that they acquire from the environment during larval development and house within specialized gill cells (11–13). These chemosynthetic symbionts provide their host with organic carbon fixed through the Calvin–Bassham–Benson (CBB) cycle, which they power by oxidizing reduced sulfur compounds from the environment (14, 15). Recent studies have revealed a surprisingly broad range of symbiont metabolic capabilities including nitrogen fixation and the capacity to grow on reduced one-carbon compounds (16). This large repertoire of metabolic functions may be critical to their survival under the contrasting conditions of their symbiotic and free-living phases. Indeed, all lucinid symbionts studied to date possess functional traits typical of free-living gammaproteobacteria such as heterotrophic metabolism (15, 16). Despite our growing understanding of their metabolic capabilities, studies investigating lucinid symbiont biodiversity are scarce and limited in their taxonomic and geographic scope. Lucinidae is the most species-rich family of chemosymbiotic bivalves, comprising roughly 400 species that thrive in a wide array of shallow and deep-water marine habitats, which suggests a great diversity of lucinid symbionts remains to be discovered (17). This diversity of hosts and habitats, distributed throughout the globe, makes lucinids a powerful system for unraveling the ecological, biogeographic, and systematic factors influencing diversity and flexibility in horizontally transmitted nutritional symbioses.

We used deep-coverage metagenomics to study diversity and flexibility in the association between lucinids and their chemosynthetic symbionts, focusing on populations and species in the Caribbean and the Mediterranean. We report a lucinid symbiont—*Candidatus* Thiodiazotropha taylori—associated with eight host species, representing three lucinid subfamilies, from distant locations across the globe. Our findings suggest a high degree of flexibility in partner choice could be an important factor in the ecological and evolutionary success of the lucinid symbiosis. We further examined how homologous recombination has shaped symbiont biology and carried out comparative genomics to investigate the functional variability among symbiont species across different host populations and species. Finally, we discuss how both symbiotic and free-living life stages influence the biology of the symbionts and their lucinid hosts.

## Results

### Individual Lucinid Hosts Can Harbor More Than One Symbiont Species Simultaneously.

We sequenced, assembled, and binned metagenomes from 47 individual clams. This resulted in 63 MAGs (metagenome assembled genomes), 53 of which were considered high-quality with greater than 90% completeness and less than 10% contamination (18) (Dataset S1). All newly assembled MAGs from this study are available under GenBank accession numbers SAMN16825223 to SAMN16825285, including raw reads (SRA database project number PRJNA679177). All 63 MAGs were assigned to the genus *Ca.* Thiodiazotropha. Sixteen metagenomes yielded two distinct *Ca*. Thiodiazotropha MAGs in one gill. The majority of lucinids we investigated hosted a single symbiont species abundant enough for its genome to be assembled from gill metagenomes. Of these, 15 *Clathrolucina costata* and 1 *Loripes orbiculatus* contained two distinct MAGs (Dataset S1). Using DESMAN, no more than two strains were detected within any MAG, indicating that there is also limited symbiont strain diversity in each host individual (Dataset S1).

### Symbionts Form Distinct Clades within the Gammaproteobacterial Family Sedimenticolaceae.

**Fig. 1.**
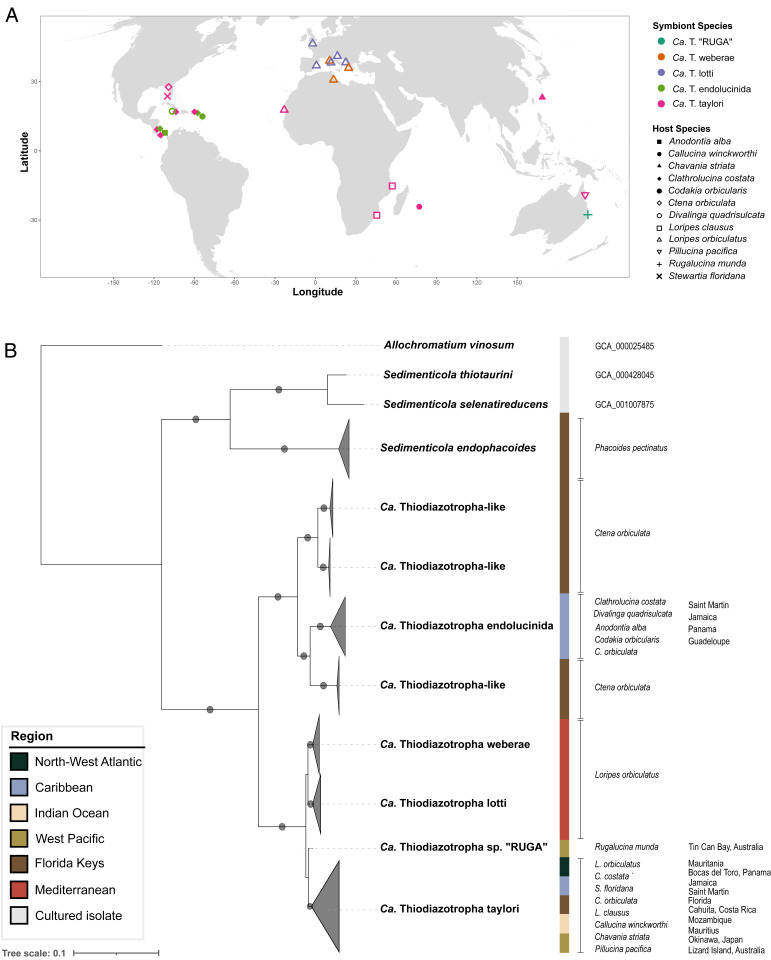
Geographic distribution and host specificity of lucinid symbiont species. (*A*) Global biogeography of symbiont species in the genus *Ca.* Thiodiazotropha reveals both localized (Mediterranean and Caribbean) and globally distributed symbiont groups. Shapes represent host species and colors represent symbiont species. *Ca*. T. taylori (pink) was found in association with eight lucinid species across the globe. *Ca.* T. sp. “RUGA” (teal) is the endosymbiont of a *R. munda* specimen from Tin Can Bay, Queensland, Australia. *Ca*. T. endolucinida (green) is distributed throughout the Caribbean and also associates with multiple host species. *Ca*. T. endoloripes, previously described as a single species by Petersen et al. (15), is in fact two closely related species (*Ca*. T. weberae in orange and *Ca*. T. lotti in purple), so far found exclusively within *L. orbiculatus* in the Mediterranean. (*B*) Phylogenetic relationships of lucinid endosymbionts. Shown is a maximum likelihood phylogenetic tree reconstructed from 43 conserved marker genes. Circles indicate bootstrap support values above 95%. Colors indicate geographic origin of the sample.

We reconstructed the phylogenetic relationships of 63 *Ca*. Thiodiazotropha MAGs we generated to previously described lucinid symbionts and their closest free-living relatives, *Sedimenticola thiotaurini* and *Sedimenticola selenatireducens*, using a concatenated amino acid alignment of 43 universally conserved marker genes, with *Allochromatium vinosum* as an outgroup [alignment on Figshare (19)]. The maximum likelihood phylogenetic reconstruction yielded 10 unique clades, each with an average nucleotide identity (ANI) above 95%, indicating that each clade likely corresponds to a distinct species, two of which have not been previously identified (Dataset S2). Apart from the symbionts of *Phacoides pectinatus*, *Ca.* Sedimenticola endophacoides, all other lucinid symbionts belong to a clade that most likely represents a single genus, named *Ca.* Thiodiazotropha. The first of the two species we identified was represented by 36 MAGs assembled from lucinid hosts from across the Pacific, Atlantic, and Indian Oceans, making it the most widely distributed lucinid symbiont known to date. We propose the name *Candidatus* Thiodiazotropha taylori for this species after Dr. John Taylor (*SI Appendix*, *Supplementary Discussion*), to honor his enormous contribution to understanding the biodiversity and evolution of the Lucinidae. *Ca*. T. taylori was associated with eight host species from three different subfamilies of Lucinidae (Lucininae, Leucosphaerinae, and Codakiinae), making this the most diverse group of hosts that any lucinid symbiont species is known to associate with (Fig. 1). The second species, *Ca*. T. sp. “RUGA,” was found in a single *Rugalucina munda* metagenome. The *Ca*. T. sp. “RUGA” MAG shared an ANI of 94.543% with all *Ca.* T. taylori MAGs, making it the closest identified relative to the *Ca*. T. taylori group.

**Fig. 2.**
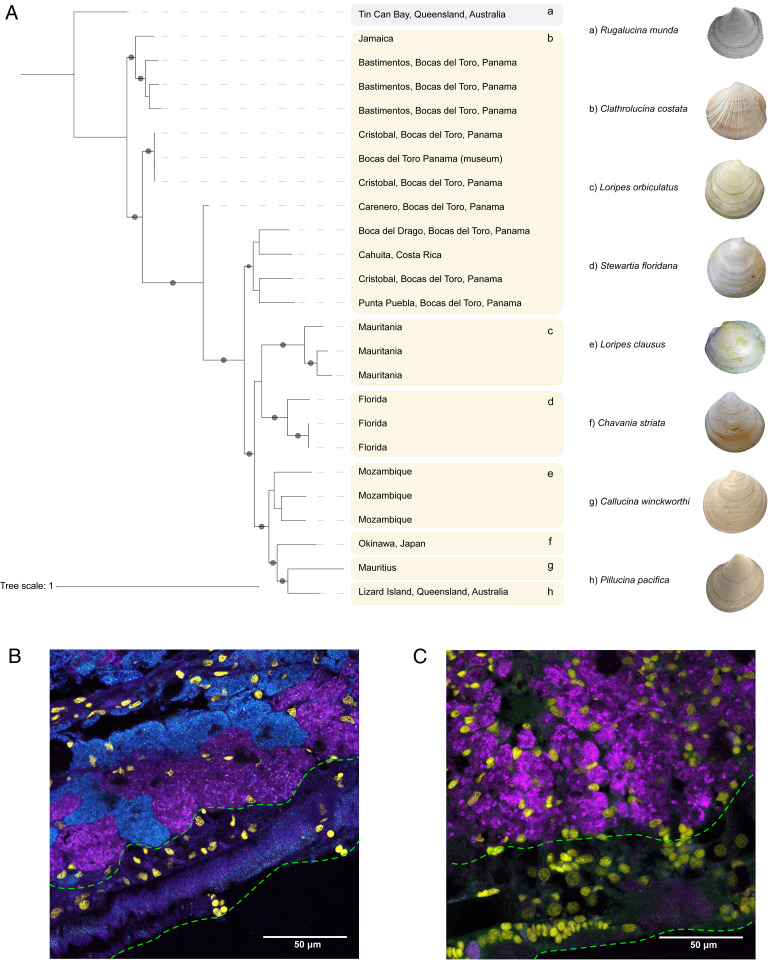
Phylogenetic relationships of *Ca*. Thiodiazotropha taylori MAGs and distribution across host species. (*A*) Phylogenetic relationship among *Ca*. T. taylori MAGs (high quality) and the individual host species where these MAGs were found in (b–h; a = outgroup symbiont found in the host *Rugalucina munda* colored in gray). The core gene alignment of *Ca*. T. taylori was constructed by aligning all shared genes in progressiveMauve and correcting for recombination in ClonalFrameML (*SI Appendix*, Fig. S1). This corrected alignment [alignment available on Figshare (90)] was uploaded to the IQtree web interface to construct a maximum likelihood phylogenetic tree with 1,000 bootstraps. The best-fit substitution model was TVM+F+ASC according to BIC scoring. Circles indicate support values above 95%. Note that *Ca*. T. taylori was found in eight host species but one host MAG was found to have low completion and high contamination and was therefore not included in this analysis (*C. orbicularis*, Florida). Shell images reprinted with permission from ref. 91. Lucinid shells are not to scale. (*B*) Spatial distribution of *Ca*. T. taylori in the gills of *C. costata* and (*C*) *L. orbiculatus.* Magenta, *Ca*. T. taylori; cyan, T. endolucinida; yellow, DAPI-labeled nuclei; green dashed lines, zone of ciliated epithelial cells.

Reanalysis of *L. orbiculatus* metagenomes containing a previously described symbiont species, *Ca.* T. endoloripes, revealed that *Ca.* T. endoloripes is actually two distinct lineages with an ANI of 91.938%, which indicates they are separate species (Dataset S2). We propose the names *Ca*. T. weberae and *Ca*. T. lotti for these two species after Dr. Miriam Weber and Christian Lott, who discovered the population of *L. orbiculatus* in Elba, Italy. Its symbionts *Ca*. T. weberae and *Ca*. T. lotti both share an ANI of roughly 89% with *Ca.* T. taylori and *Ca*. T. sp. “RUGA,” their next-closest relatives (Fig. 2). The lucinid symbionts from the genus *Ca*. Thiodiazotropha formed two major clades, one comprising the four above-mentioned species and another containing *Ca*. T. endolucinida and the Thiodiazotropha-like species associated with *Ctena orbiculata* from Florida, previously reported by Lim et al. (20) (Fig. 2). Like *Ca.* T. taylori, *Ca*. T. endolucinida was also found in multiple host species (five species from subfamilies Lucininae, Codakiinae, and Leucosphaerinae) but its geographic distribution appears to be restricted to the Caribbean based on samples that are so far available (February 2021).

### Comparative Genomics of Lucinidae Symbionts.

We compared the predicted functional capabilities of *Ca*. T. taylori, the most widespread symbiont in our dataset, to 1) its closest relatives (*Ca*. T. weberae, *Ca*. T. lotti, and *Ca*. T. sp. “RUGA”) and 2) the sympatric *Ca*. T. endolucinida, which can cooccur with *Ca*. T. taylori in the same host gill, to investigate whether functional potential reflects symbiont geography or phylogeny. All five symbiont species shared most core metabolic functions (Table 1). A maximum of 7.2%, or 170 of the 2,361 protein families (Pfams) found in these genomes, were enriched in *Ca*. T. endolucinida, i.e., they were significantly more frequent within this species group (*P*_*adj*_ < 0.05), while the closely related species *Ca*. T. taylori, weberae, and lotti had far fewer enriched Pfam functions, suggesting that phylogenetic relationships are to some extent reflected in genomic functional potential (details in *SI Appendix*, *SI Results*, Table S3, and Fig. S2 *A* and *B* and Dataset S5). In other words, the more closely related the symbionts, the more likely they are to share genome content.

**Table 1.** Comparison of the predicted major metabolic functions annotated in the MAGs of *Ca.* T. taylori, *Ca.* T. weberae, *Ca.* T. lotti, *Ca.* T. sp. “RUGA,” and *Ca.* T. endolucinida

Feature	*Ca*. T. taylori	*Ca*. T. endolucinida	*Ca*. T. weberae	*Ca*. T. lotti	*Ca*. T. sp. “RUGA”
Carbon metabolism					
CBB cycle, form I (RuBisCO)	+	+	+	+	+
CBB cycle, form II (RuBisCO)	−	+	−	−	−
Methylotrophy pathway*	+	+	−	−	+
Nitrogen metabolism					
Diazotrophy, nitrogenase	+	+	+	+	+
Respiratory nitrate reductase	−	+	−	−	−
Copper-containing nitrite reductase (NO-forming)	−^†^	+	−	+	+
Nitric-oxide reductase	+	+	+	+	+
Nitrous-oxide reductase	+	+	+	+	+
Periplasmic nitrate reductase	+	+	+	+	+
Nitrite reductase NADPH subunit	+	+	+	+	+
Urease	+	−	+	−	−
Ammonia assimilation	+	+	+	+	+
Sulfur metabolism					
Sqr	+	+	+	+	+
Truncated SOX	+	+	+	+	+
DSR	+	+	+	+	+
DsrMKJOP complex	+	+	+	+	+
APR	+	+	+	+	+
FCC	+	+	+	+	+

+, gene(s) within the pathway were present in all the high-quality MAGs; −, gene(s) within the pathway were absent from all the high-quality MAGs. Further details are available in *SI Appendix*, Table S2 and Dataset S3.

* This cluster of genes is putatively annotated with the function of methylotrophy. Note that a recent study implicated similar genes in tetrathionate oxidation (92). Further studies are required to elucidate their true function.

^†^ These genes were only present in the high-quality MAGs of *Ca*. T. taylori associated with *Stewartia floridana*, from Florida.

### Core Metabolic Pathways Shared by All Symbiont Species.

The *Ca*. Thiodiazotropha symbionts rely on multiple pathways to oxidize reduced sulfur compounds to sulfate through a polysulfur or elemental sulfur intermediate and there were no major variations in sulfur oxidation capabilities across all the MAGs (Table 1 and *SI Appendix*, Table S2 and Dataset S3). The energy generated from sulfur oxidation is used to power inorganic carbon fixation through the CBB cycle (Table 1). The MAGs of *Ca*. T. taylori, T. weberae, T. lotti, T. sp. “RUGA,” and T. endolucinida each contained a gene encoding Ribulose-1,5-bisphosphate carboxylase/oxygenase (RuBisCO) form I (Table 1). *Ca.* T. endolucinida was the only species that possessed two distinct types of RuBisCO, forms I and II. All five species also encoded the capacity for heterotrophic growth (Table 1). The genus name *Thiodiazotropha* was proposed following the recent discovery that lucinid symbionts are capable of fixing inorganic nitrogen from the atmosphere (14, 15). Indeed, all five symbionts encoded large gene clusters involved in nitrogen fixation, including the widely used functional marker for nitrogen fixation, dinitrogenase reductase subunit (*nifH*), the structural genes (*nifD* and *nifK*), ferredoxins, and regulatory factors (Dataset S3). All species had a complete denitrification pathway for reducing nitrate and nitrite to nitrogen gas, and thus all had the potential to use nitrate and nitrite as alternative electron acceptors (Table 1).

### Accessory Metabolic Functions in Carbon and Nitrogen Metabolism.

Despite sharing most metabolic functions that sustain the nutritional lucinid symbiosis, we observed notable variations in symbiont carbon and nitrogen metabolic pathways. *Ca.* T. taylori, *Ca.* T. endolucinida, and *Ca.* T. sp. “RUGA” possessed genes required for growth on reduced one-carbon compounds (20), including lanthanide-dependent methanol dehydrogenase (*xoxF*-like), genes for the synthesis of its cofactor pyrroloquinoline quinone (*pqqABCDE*), and the full tetrahydromethanopterin-dependent pathway for formaldehyde oxidation, which was found in close proximity to methanol dehydrogenase (21, 22). The presence of this cluster of genes suggests these symbionts are able to convert methanol to aldehydes, which can be subsequently converted to biomass or utilized as energy. All five species were capable of assimilating nitrogen gas and ammonia but there were notable differences in their ability to assimilate urea (Table 1), a potential waste product of lucinid clams. Only MAGs of *Ca.* T. taylori and *Ca.* T. weberae encoded genes for the uptake and conversion of urea to ammonia (*ureACDEFGJ*). MAGs of *Ca.* T. taylori, *Ca.* T. lotti, *Ca.* T. weberae, and *Ca.* T. “RUGA” also encoded genes for an alternative pathway for urea metabolism through urea amidolyase (*SI Appendix*, Table S2). Finally, *Ca.* T. endolucinida was the only species with MAGs encoding genes for the respiratory nitrate reductase complex (*narGHJI*), indicating that it can reduce nitrate for generating a proton motive force. These observations were based on the presence/absence of entire operons/gene clusters and were consistent across all the high-quality MAGs examined, of which we had multiple samples from each symbiont species (minimum of nine).

### Recombination Rates.

Nucleotide differences in closely related bacterial sequences can be caused by two primary mechanisms, either homologous recombination (HR), where a fragment of the recipient’s genome is replaced by that of a donor in a single generation (genetic exchange), or by mutations from one generation to the next (23). In the absence of HR, all differences in genomic sequences reflect a clonal genealogy. We calculated average HR to mutation rates (R/m) for each species group and for groups containing multiple species. The alignment lengths of shared genes used to calculate R/m ranged from 1,751,700 to 3,149,280 base pairs (bp), which for some groups covered more than half of the total length of the MAGs (Table 2). The alignment of shared genes was shortest in *Ca.* T. taylori due to the strict alignment parameters chosen (i.e., aligned genes must be present in all samples) and a greater genetic diversity within this species group. Just as *Ca.* T. taylori is unique among lucinid symbiont species for its extensive geographic range, its average R/m ratio was at least 10 times higher than the average R/m ratios in all the other species groups (0.814; 0.809 to 0.819; 95% confidence intervals; Table 2). The average R/m ratios for *Ca*. T. taylori populations from different locations were fairly consistent and ranged from 0.859 in Florida to 1.521 in Mauritania (*SI Appendix*, Table S4). The lowest R/m values were between *Ca*. T. endolucinida and *Ca*. T. taylori (0.053; 0.052 to 0.054), even though these can cooccur within host individuals. The lengths of all alignments, average number of recombination events, average length of events, and all R/m ratios and their 95% confidence intervals are shown in *SI Appendix*, Table S4. The average nucleotide diversity outside the recombining gene sequences was 3.6, 2.4, 1.9, and 0.6 single-nucleotide variants per kbp for *Ca*. T. taylori, *Ca*. T. lotti, *Ca*. T. weberae, and *Ca*. T. endolucinida, respectively (Dataset S6). Although MAGs consist of consensus sequences of the most abundant representatives of an entire bacterial population, the low strain estimates from DESMAN (two or fewer strains) across all MAGs in this study provide confidence that these results are not confounded by strain differences within the gill metagenomes (*SI Appendix*, Table S1).

**Table 2.** Overall statistics from individual species and cooccurring pairs in ClonalFrameML

Species	Length of genome alignment, bp	Recombination to mutation ratio (95% CI)
*Ca.* T. taylori	1,751,700	0.814 (0.809–0.819)
*Ca.* T. weberae	3,149,280	0.043 (0.041–0.046)
*Ca.* T. lotti	3,149,280	0.085 (0.083–0.087)
*Ca.* T. endolucinida	3,420,600	0.082 (0.079–0.084)
Cooccurring pairs		
* Ca*. T. taylori–T. endolucinida	1,664,616	0.0066 (0.0063–0.0069)
* Ca*. T. weberae–T. lotti	3,149,280*	0.053 (0.052–0.054)

* The same core gene alignment was used for *Ca*. T. weberae and T. lotti together as well as each one individually.

## Discussion

### A Single Cosmopolitan Symbiont Species Associates with Multiple Diverse Lucinid Host Species at Locations around the World.

We investigated the diversity and the predicted functional variability of the symbionts associated with lucinids on a global scale. Fresh samples were complemented with specimens from museum collections to expand the scope of the study beyond the most intensively studied lucinid habitats in the Caribbean and Mediterranean Seas (15, 20, 24). We identified two lucinid symbiont species, *Ca*. T. taylori and *Ca*. T. “RUGA.” *Ca*. T. taylori is found in a remarkable eight different lucinid species from three different subfamilies (Figs. 1 and 2), which makes *Ca*. T. taylori the most promiscuous lucinid symbiont described thus far and the first chemosynthetic endosymbiont species with a globally distributed population (25, 26). We similarly found *Ca*. T. endolucinida in five divergent host species from three distinct lucinid subfamilies (Fig. 1). These findings corroborate previous reports that the same symbiont 16S ribosomal RNA (rRNA) gene sequence, identical to that of *Ca*. T. endolucinida (*SI Appendix*, Fig. S3), was present in four distinct host species at one location in Guadeloupe (25). We predict that future surveys of lucinid symbiont diversity are likely to reveal similar instances of promiscuity. The remarkable flexibility in the association between lucinids and *Ca*. Thiodiazotropha species could thus be an important feature underlying the evolutionary success of this ancient and widespread symbiosis.

### HR Maintains the Cohesiveness of *Ca*. T. taylori as a Single “Species.”

Genetic exchange plays a critical role in bacterial genome evolution and can either drive the divergence or homogenization of a population (27). HR drives homogenization by maintaining genomic cohesion of bacterial clades even across distant locations (28, 29). We reconstructed recombination events in four symbiont species groups—*Ca*. T. taylori, T. endolucinida, T. weberae, and T. lotti—to investigate the role of HR in maintaining genomic cohesion of *Ca*. T. taylori as a single species-level group. The rate of recombination to mutation (R/m) in *Ca*. T. taylori (0.814) is above the theoretical threshold for preventing population divergence (0.25) (29–31). In contrast, the R/m rates of *Ca*. T. endolucinida, T. weberae, and T. lotti are well below this 0.25 threshold (0.04 to 0.08), a pattern that correlates with the limited geographic range of these three species compared to *Ca*. T. taylori (Fig. 1). With such a large distribution range spanning many diverse habitats, selection pressures or neutral drift could cause local *Ca*. T. taylori populations to diverge, which might result in gene content and functional differences (Table 1). This is supported by our phylogenetic reconstruction of the *Ca*. T. taylori clonal frame (Fig. 2), which reveals that clams from each distinct geographic location are colonized by site-specific phylogenetic lineages that were poorly resolved in the phylogenomic tree (Fig. 1). Our recombination analyses indicate that HR has a cohesive effect that maintains the integrity of *Ca*. T. taylori as a single cosmopolitan species-level group. With an ANI of about 94.5%, the *Ca*. T. taylori and *Ca*. T. “RUGA” MAGs represent distinct, albeit closely related, genetic units (Fig. 1 and Dataset S1). Given that HR rates drop exponentially with decreasing sequence similarity, declining steeply between 90 to 95% ANI (32), we speculate that this seemingly small amount of nucleotide divergence likely prevents recombination between *Ca*. T. “RUGA” and *Ca*. T. taylori. Indeed, an ANI of 95% is widely used as a threshold for delineating bacterial species, as this is thought to correspond to a level of divergence that prevents genetic exchange by HR (33–35). One could speculate that *Ca*. T. RUGA and *Ca*. T. taylori previously belonged to a single species-level group but were relatively recently separated and that if the local geographic *Ca*. T. taylori populations encountered a barrier to HR they too might become new and distinct species-level groups. It is currently unclear which barriers could “remove” local populations from the cohesive forces of HR and allow them to diverge independently. Greater efforts to sample and sequence more *Ca*. T. “RUGA” and *Ca*. T. taylori symbionts will allow us to test these and other theories about the emergence of symbiont diversity in future.

The cosmopolitan distribution of lucinid clams hosting *Ca*. T. taylori suggests this symbiont species is able to disperse over great geographic distances. How *Ca*. T. taylori achieves this feat of dispersal remains a mystery but hitchhiking on their bivalve hosts during the planktonic larval phase appears unlikely because the lucinid larvae studied thus far have been found to be aposymbiotic (11). Furthermore, the range of this symbiont species far exceeds the limited distribution range of any lucinid host species. *Ca*. T. taylori therefore likely migrates during its free-living phase rather than its host-associated phase. The dormant endospores of thermophilic Firmicutes achieve extensive distributions through long-distance passive dispersal in oceanic currents, but even these tough endospores with enhanced survival capacities face more substantial dispersal limitations than *Ca*. T. taylori (36). It seems unlikely that free-living *Ca*. T. taylori cells would be able to survive harsh open-ocean conditions long enough to traverse the globe. To our knowledge, there are no molecular data to date indicating *Ca*. Thiodazotropha spp. are present at a high abundance in coastal sediments or the water column, which suggests they are members of the rare biosphere present only at low relative abundance (37). Troussellier et al. put forward the intriguing hypothesis that macroorganisms could sustain the rare biosphere by serving as dissemination vectors for marine microbes (38). A recent meta-analysis of publicly available amplicon sequencing data revealed the presence of *Ca.* Thiodiazatropha 16S rRNA gene sequences associated with the roots of various seagrass species around the globe, a discovery the authors subsequently verified with microscopic imaging (37). Vegetative seagrass fragments (shoots and rhizomes) can reestablish in distant locations after detaching from their parents and have great potential for long-distance dispersal (39, 40). It is interesting to speculate that lucinid clams and seagrasses from across the globe could form a network of source habitats facilitating the dissemination and dispersal of *Ca.* T. taylori vectored by oceanic circulation, thereby overcoming potential barriers to dispersal between geographically distant bodies of water.

Despite its global distribution range, we may have identified one potential barrier to dispersal of *Ca*. T. taylori. Rigorous sampling of *L. orbiculatus* along the Atlantic and Mediterranean coasts of Europe did not reveal a single instance of *Ca*. T. taylori associated with *L. orbiculatus* in any of these locations (Fig. 1). Instead, *L. orbiculatus* in these locations all hosted the closely related sister species *Ca*. T. weberae and T. lotti (Fig. 1). The colonization of *L. orbiculatus* by *Ca.* T. lotti from the United Kingdom all the way south to Kotor, Montenegro suggests the strait of Gibraltar is unlikely to pose a barrier to the dispersal of *Ca*. T. taylori. Nor are these distribution patterns driven by host-symbiont specificity, because *L. orbiculatus* in Mauritania hosts *Ca*. T. taylori (Fig. 1). A possible explanation is that environmental factors associated with a temperate climate prevent *Ca*. T. taylori from establishing in clams along the coasts of Europe. This is consistent with the tropical distribution of all lucinid species so far found to host *Ca*. T. taylori (Fig. 1). An alternate nonmutually exclusive explanation could be that *Ca*. T. weberae and T. lotti are better adapted and able to outcompete *Ca*. T. taylori for establishment in lucinid hosts throughout temperate Europe. Further sampling of other lucinid species and seagrasses along European coasts will be necessary to address these questions.

### Coexisting *Ca.* Thiodiazotropha Species Have Distinct Metabolic Capabilities.

Lim et al. (20) recently reported multiple *Ca*. Thiodiazotropha 16S rRNA gene amplicon sequence variants and MAGs of diverse *Ca*. Thiodiazotropha spp. associated with *C. orbiculata* individuals from Florida, providing the first indications that multiple symbiont species from the same genus may coexist in the same host gill. We were able to assemble and bin MAGs of two distinct symbiont species, *Ca*. T. taylori and T. endolucinida, from the metagenomes of 15 *C. costata* individuals sampled from across the Caribbean. Using the same methods, a reanalysis of new and previously published gill metagenome data (LVJZ00000000) from *L. orbiculatus* (Elba, Italy) similarly revealed that MAGs from two distinct symbiont species, *Ca*. T. lotti and T. weberae, can be assembled and binned from metagenomes of single host individuals. Our fluorescence in situ hybridization (FISH) results showed that in *C. costata*, *Ca*. T. taylori and T. endolucinida both inhabit gill epithelial cells. These findings add to a growing number of studies reporting the coexistence of closely related chemosynthetic symbiont species/strains within the same invertebrate host and indicate that this is more common in lucinids than previously assumed (41–43).

Ecological models predict that cooccurring symbionts with the same resource requirements will compete for limited resources and that this competition is detrimental to the symbiosis (e.g., ref. 44). The cooccurring symbiont pairs we identified, *Ca*. T. taylori/endolucinida and *Ca*. T. weberae/lotti, shared core metabolic functions fundamental to their symbiosis with lucinid clams (15), including identical pathways for oxidizing sulfur, fixing inorganic carbon, and fixing nitrogen. *Ca*. T. taylori and *Ca.* T. endolucinida both had the additional potential to utilize methanol as a source of energy and carbon with an *xox*-type methanol dehydrogenase and the serine pathway for C1-carbon incorporation into biomass. Compartmentalizing coexisting symbiont species into separate bacteriocytes could prevent direct competition by allowing the host to partition resources and discriminate cooperative symbionts from potential cheaters that might destabilize the symbiosis (45). Consistent with this, our FISH analyses showed that although symbiont species cooccurred in host individuals they never cooccurred in single host bacteriocytes (Fig. 2). Despite their prolific productivity, habitats abundant with lucinids, such as seagrass meadows and coral reef lagoons, tend to occur in oligotrophic waters that are nitrogen-limited (14). It is noteworthy that the ability to hydrolyze urea to ammonia, which can subsequently be assimilated, is a conserved function of *Ca*. T. taylori and *Ca*. T. weberae that is absent in *Ca*. T. endolucinida and *Ca*. T. lotti (Table 1). The precise composition of lucinid nitrogenous waste remains unknown, but some bivalves do excrete urea as a waste product and the urea transporter DUR3 is highly expressed in the gills of *L. orbiculatus* (46, 47). Future studies are required to investigate whether the ability to utilize urea as a nitrogen source confers any advantage to *Ca*. T. taylori and *Ca*. T. weberae in the host or the external environment.

We observed some additional predicted metabolic differences between *Ca*. T. taylori and *Ca*. T. endolucinida. This symbiont pair has one major difference: *Ca*. T. endolucinida MAGs encode both RuBisCo forms I and II, while *Ca*. T taylori only encoded form I (Table 1). *Ca*. T. endolucinida also has the genes encoding a respiratory nitrate reductase protein complex, which indicates the ability to use nitrate instead of oxygen as an electron acceptor for respiration (Table 1). This combination of functional traits suggests *Ca*. T. endolucinida may be better adapted to survival in a lower-oxygen environment than *Ca*. T. taylori. This could be highly beneficial for the symbiosis as the endosymbionts of both the lucinid clam *Lucinoma aequizonata* and the vent tubeworm *Riftia pachyptila* are able to respire nitrate as an adaptation to living in deep-sea environments with fluctuating oxygen availability (48–50). Whether shallow-water lucinids in the Caribbean are able to exploit this unique metabolic capability of *Ca*. T. endolucinida remains unknown but there are several observations suggesting it does not. First, the vanishingly small nitrate reductase activity in the gills of *Codakia orbicularis*, an established host of *Ca*. T. endolucinida, and the absence of nitrates in the tissues of this host species together suggest nitrate is not used as an electron acceptor in this symbiosis (51). Second, nitrate is only sporadically present at low concentrations in the pore water of *Thalassia testudinum* sediments and is undetectable in the overlying waters (14, 51). Third, oxygen is abundantly available in Caribbean lucinid habitats, especially during periods of photosynthesis, and lucinid clams construct burrows leading to the surface to obtain oxygenated water from above the sediment layer (52). These observations are inconsistent with *Ca*. T. endolucinida’s requiring these metabolic adaptations while they are housed in the buffered environment of the lucinid bacteriocytes. Moreover, our analysis of the *C. orbicularis* metatranscriptome indicates that the genes encoding RuBisCO form II and respiratory nitrate reductase are expressed at a much lower level compared to RuBisCo form I and the assimilatory nitrate reductases, respectively (Dataset S7). These data are further supported by the much lower abundance of RuBisCo form II proteins compared to form I in the *C. orbicularis* proteome (14). Based on these observations, we hypothesize that *Ca*. T. endolucinida relies on these metabolic functions primarily during its free-living phase and we speculate that *Ca*. T. endolucinida could occupy an external environmental niche—one characterized by low oxygen availability— distinct from that of *Ca*. T. taylori.

## Materials and Methods

### Sample Collection.

Live clams were collected from sites throughout the Caribbean and Mediterranean (*SI Appendix*, *SI Methods* and Dataset S1). Gills were dissected in the field and preserved in RNAlater (AM7020; Life Technologies) or DNA/RNA Shield (R1100-250; ZymoBiomics) according to manufacturer’s instructions and stored at −20 °C. Samples from locations in the Pacific and Indian Oceans came from the collections of the Natural History Museum (NHM) in London and the Florida Natural History Museum (FLMNH), Gainesville, FL (*SI Appendix*, *SI Methods* and Dataset S1). Access was permitted and organized by Dr. John Taylor (NHM) and Dr. Gustav Paulay and Dr. Amanda Bemis (FLMNH). *SI Appendix*, *SI Methods* and Dataset S1 list all the species used, sampling locations, dates, and sample sizes.

### DNA Extraction, Preparation, and Sequencing.

DNA was extracted from gill tissues using the Qiagen DNeasy Blood and Tissue kit (69506; Qiagen) or the animal tissue protocol from Analytikjena Innuprep DNA Mini Kit (845-KS-1041250) (*SI Appendix*, *SI Methods*). Samples were either treated with RNase or directly quantified before DNA libraries were prepared using Illumina-compatible library prep kits (Dataset S1 and *SI Appendix*, *SI Methods* and Table S5). All libraries were sequenced with Illumina technology to generate paired-end reads of 150 bp or 250 bp length (*SI Appendix*, *SI Methods* and Dataset S1).

### Quality Filtering, Assembly, and Bacterial Genome Binning.

Read libraries were trimmed, PhiX contamination-filtered, and quality-checked using BBMAP v37.61’s BBDUK feature (53); parameters used are in *SI Appendix*, *SI Methods* and the Jupyter notebook. Individual read libraries were assembled using SPAdes v3.13.1 (54, 55); parameters used are in *SI Appendix*, *SI Methods*. The resulting metagenomic assembly scaffolds were binned using a combination of Anvi'o v6.1 (56, 57) using CONCOCT v1.1.0 (58) and MetaBAT v2.15 (59) (details and parameters in *SI Appendix*, *SI Methods* and Dataset S1). The bins were then compared using dRep v2.4.2’s dereplicate workflow (60) (*SI Appendix*, *SI Methods*). The bins were checked for completion using Checkm’s taxonomy specific workflow and manually refined using “anvi-refine” (*SI Appendix*, *SI Methods*). MAGs that were determined to be 90% or more complete and less than 10% contaminated post refinement, referred to as high-quality MAGs, were used for further analyses. Potential strain numbers in individual clams/metagenomes were calculated using DESMAN v2.1.1 (61) and the snakemake workflow (62) on the program (*SI Appendix*, *SI Methods*).

### Phylogenetic Analyses.

Only the MAGs taxonomically assigned to Sedimenticolaceae or Chromatiaceae, using GTDB v0.3.3 (63) were used in this study. We also downloaded publically available MAGs of other lucinid symbionts (*Ca*.Thiodiazotropha spp. and *Sedimenticola spp*.), alongside *A. vinosum* as an outgroup, for this analysis (complete list of accession numbers in Dataset S1). The publicly available MAGs were quality-checked and filtered using the methods described above (and in *SI Appendix*, *SI Methods*). The CheckM v1.1.3 (64) lineage workflow was used to identify, align, and concatenate a default set of 43 universal marker genes from all the available MAGs (Dataset S1); concatenated marker gene alignment can be found on Figshare (19). This concatenated amino acid alignment was then submitted to the W-IQ-TREE server (65) using default settings (*SI Appendix*, *SI Methods*) and the resulting maximum likelihood tree was visualized using the Interactive Tree Of Life (iTOL) v5 (66). All MAGs were placed into species groups based on ANI values above 95% using FastANI v1.3 (34). The accuracy of these species boundaries was tested with ANIb and ANIm through the jspecies web server (67).

### Localization of Symbionts in Clam Gills.

We carried out FISH to visualize *Ca*. T. taylori in gill sections of *L. orbiculatus* (Mauritania, 2018) and *C. costata* (Panama, 2019). Probes were designed to target the 16S rRNA gene sequence of *Ca*. T. taylori using DECIPHER’s design probes web tool (*SI Appendix*, *SI Methods*) (68). A formamide series from 0 to 60%, in 10% steps, was carried out to optimize the probe hybridization conditions (*SI Appendix*, *SI Methods*; probe sequences used are in *SI Appendix*, Table S6). Nonsense sequences of the target-specific probes were also tested as negative controls (69). Dissected gills were preserved in 4% paraformaldehyde, dehydrated into 70% ethanol, and stored at 4 °C (*SI Appendix*, *SI Methods*). The gills were embedded in paraffin wax by the Histopathology Facility at Vienna BioCenter Core Facilities, Austria (*SI Appendix*, *SI Methods*). We cut the embedded gills into 5-µm sections with a Leica microtome and mounted the sections on SuperfrostPlus adhesion slides (Thermo Scientific). The sections were dewaxed in Roti-Histol (Carl Roth) following the manufacturer’s instructions. Probes were hybridized to the gill sections in a 50% formamide buffer (details on hybridization conditions and washing protocols are in *SI Appendix*, *SI Methods* and Tables S6 and S7). Following the hybridization, the samples were DAPI-stained (1 µg/mL) and mounted in ProLong Glass antifade mounting media (Thermo Fisher Scientific) (*SI Appendix*, *SI Methods*). Images were captured on a Leica TCS SP8 X confocal laser scanning microscope using a 63× objective (*SI Appendix*, *SI Methods*).

### Functional Annotation and Pangenomic Analysis of Bacterial Genomes.

Anvi’o’s pangenomic workflow was used for orthologous group clustering and functional comparisons (*SI Appendix*, *SI Methods*). We annotated all features containing open reading frames (ORFs) using eggNOG-mapper v2 (70) with eggNOG database v5.0 (71). All ORFs were also annotated with Pfam domains (72) using HMMER v3.3 (73) (parameters in *SI Appendix*, *SI Methods*). All high-quality MAGs were also used to create pangenome in Anvi’o with an mcl inflation value of 8; pangenomic genomes database and profiles are on Figshare (74, 75). The MAGs were also annotated on the Rapid Annotation using Subsystem Technology (RAST) web server (https://rast.nmpdr.org/) using the RASTtk pipeline (76). Where necessary, genes of interest were manually screened using NCBI blast+ v2.8.1 (77) and BBMAP to search for genes potentially missing from the assemblies (*SI Appendix*, *SI Methods*). To determine if any annotated functions were present in a given species group or population at a higher frequency than expected under a uniform distribution, EggNOG and Pfam terms were statistically tested for enrichment across different species groups and populations through the “anvi-get-enriched-functions-per-pan-group” function in Anvi’o with an “adjusted q value” cutoff of 0.05 (*SI Appendix*, *SI Methods*).

### *C. orbicularis* Metatranscriptome Analysis.

Three *C. orbicularis* specimens were collected in Guadeloupe (2016), preserved in RNAlater, and stored at −80 °C. Total RNA was extracted using TRIzol (Thermo Fisher Scientific) according to the manufacturer's instructions. rRNA depletion and library construction were carried out at the Vienna Biocenter Core Facilities GmbH as described in Yuen et al. (47). The RNA-sequencing reads were trimmed and processed as previously described by Yuen et al. (47). We used BBMAP (slow = t, ambiguous = best, minid = 0.99) to align the processed reads to the MAG of *Ca*. T. endolucinida (GCA_001715975.1), an endosymbiont of *C. orbicularis* from Guadeloupe (53). FeatureCounts was used to quantify gene-level transcript abundances, which were subsequently converted to transcripts per million (78). Metatranscriptomic results can be found in Dataset S7.

### Recombination Rates and Nucleotide Diversity of Bacterial Symbionts.

To infer recombination events in bacterial genomes, we used the maximum likelihood implementation of ClonalFrame, ClonalFrameML (23). High-quality MAGs were aligned using progressiveMauve, in Mauve v2.0 (79). Nucleotide sequences (i.e., core genes) shared within the groups of MAGs analyzed were extracted with stripSubsetLCBs, a script previously described in ref. 80. These core genes were realigned with MUSCLE (81) and cleaned with trimAl (82) (parameters in *SI Appendix*, *SI Methods*). RAxML v8.2.10 (83) was used to build a phylogenetic tree from this new alignment as described in ref. 84. All alignments are available on Figshare (85–87). The resulting tree and alignment were fed into ClonalFrameML to calculate the ratio of recombination vs. mutation events with default parameters (See Dataset S1 for MAGs used in this analysis). Recombination events were computed and visualized in R v6.3.2 with the script cfml_results.R (*SI Appendix*, *SI Methods*). GUBBINS was used to estimate average nucleotide diversities inside and outside the clonal frame (88). We also used the clonal frame alignments to reconstruct and visualize the phylogenetic relationships of the *Ca*. T. taylori MAGs as previously described (*SI Appendix*, *SI Methods*). All scripts used can be found in the associated Jupyter Notebook (89).

## Supplementary Material

Supplementary File

Supplementary File

Supplementary File

Supplementary File

Supplementary File

Supplementary File

Supplementary File

Supplementary File

## Data Availability

The data (raw reads, metagenomic assemblies, and MAGs) have been deposited with links to BioProject accession number PRJNA679177 in the NCBI BioProject database (https://www.ncbi.nlm.nih.gov/bioproject/). The BioSample accession numbers for the MAGS are SAMN16825223–SAMN16825285 and SAMN16952162–SAMN16952207 are the corresponding raw read sets. Analysis scripts are available at https://doi.org/10.6084/m9.figshare.13295912.v5. Datasets are available on Figshare at https://figshare.com/projects/A_globally_distributed_symbiont_challenges_host_specificity_in_lucinid_clams/93398. All other study data are included in the article and/or supporting information.
