# Supplementary material for: Global biogeography of chemosynthetic symbionts reveals both localized and globally distributed symbiont groups
Source: Proc Natl Acad Sci U S A. 2021 Jul 16;118(29):e2104378118. doi: 10.1073/pnas.2104378118 (PMC8307296; doi:10.1073/pnas.2104378118)
Supplement: Supplementary File [file pnas.2104378118.sapp.pdf]

PNAS
www.pnas.org

Supplementary Information for
Global biogeography of chemosynthetic symbionts reveals both localized and globally-distributed symbiont groups

Jay T. Osvatic[1,†,*], Laetitia G. E. Wilkins[2,3,†], Lukas Leibrecht[1], Matthieu Leray[4], Sarah Zauner[1], Julia Polzin[1], Yolanda Camacho[5], Olivier Gros[6], Jan A. van Gils[7], Jonathan A. Eisen[2,8,9], Jillian M. Petersen[1,‡], and Benedict Yuen[1,‡,*]

Corresponding authors: Benedict Yuen and Jillian Petersen
Email: benedict.yuen@univie.ac.at and jillian.petersen@univie.ac.at

This PDF file includes:

Supplementary text
Figures S1 to S3
Tables S1 to S8
Legends for Datasets S1 to S7
SI References

Other supplementary materials for this manuscript include the following:

Datasets S1 to S7

Supplementary Information Text

SI Methods

Fresh clams

Live clams were collected from seagrass beds *(Thalassia* spp. or *Zostera marina*, Dataset S1) from Cahuita National Park, Costa Rica (2019), Bocas del Toro, Panama (2019), Discovery Bay, Jamaica (2019), Banc d'arguin, Mauritania (2015-2017), Elba, Italy (2018), Ksamil, Albania (2017), Kotor Bay, Montenegro (2019), Piran, Slovenia (2016), and Sant Carles de la Rapita, Spain (2019). Gills were dissected in the field and stored in RNAlater (Cat. No. AM7020; Life Technologies, USA) or DNA/RNA Shield (Cat. No. R1100-250; ZymoBiomics, USA) and stored at 4°C overnight and then frozen at -20°C until extraction.

Museum samples

Samples from museum collections were used to increase geographic coverage and included samples from Australia (2008), Mozambique (2006 and 2011), England (2005), Mauritius (2015), Tunisia (2009) and Japan (2003; see Dataset S1 for individual samples). These samples came from collections of the Natural History Museum (NHM) in London, UK and the Florida Natural History Museum (FLMNH), Gainesville, FL, USA. Access was permitted and organized by Dr. John Taylor at the NHM and Dr. Gustav Paulay and Dr. Amanda Bemis at the FLMNH. These samples had been stored for long-term in 100% EtOH at between 13-25°C. Gill segments were extracted using sterile scalpels and the remaining material from each of these samples was sent back to the collections.

DNA extraction, preparation, and sequencing

DNA was extracted from the gill tissues of ten lucinid species collected from the Caribbean Sea, the Mediterranean Sea, the Western Pacific, the Eastern Atlantic and the Western Indian Ocean; *Anodontia alba* (Link, 1807) n=1, *Callucina winckworthi* (Viader, 1951) n=1, *Chavania striata* (Tokunaga, 1906) n=1, *Clathrolucina costata* (d'Orbigny, 1845) n=18, *Ctena orbiculata* (Montagu, 1808) n=1, *Divalinga quadrisulcata* (d'Orbigny, 1845) n=1, *Loripes clausus* (Philippi, 1848) n=4, *Loripes orbiculatus* (Poli, 1795) n=18, *Pillucina pacifica* (Glover & Taylor, 2001) n=1, and *Rugalucina munda* (Adams, 1856) n=1 (Dataset S1).

Gills were laterally cut into 2 mm slices. Total DNA was extracted from a 1 cm^2 piece of the gill middle, weighing approximately 25 mg, using the Qiagen DNeasy Blood and Tissue kit (Cat. No. 69506; Qiagen, USA) or the animal tissue protocol from Analytikjena Innuprep DNA Mini Kit (Cat. No. 845-KS-1041250, Germany) following the manufacturer's instructions. Tissues were incubated until completely digested (2-20 hours). DNA was eluted in 50 µl of ultrapure water (Ref. 10977-015; Invitrogen, Life Technologies, USA). Samples were either treated with RNAase or directly quantified (Tables S2 and S21). For those that were treated with RNAase, RNA was digested using 1 µl of RNAse (Ref. 10036306, New England Biolabs, USA), 4 µl of buffer 1X NE buffer (New England Biolabs), and 40 µl of eluted DNA. Reactions were incubated for 20 min at 30 °C then heated to 70 °C for 15 min. RNA-free samples were purified twice with magnetic beads (Ref. No. A63881; Beckman Coulter, USA) following the manufacturer's protocol for PCR cleanup. Extracted DNA was quantified by following the manufacturer protocol for the Qubit dsDNA HS Assay Kit (Thermo Fisher Scientific, USA).

Sequencing DNA libraries were prepared using Illumina compatible library prep kits (NEBNext Ultra II FS DNA Library Prep Kit, NEBNext Ultra DNA v2 Library Prep Kit, Nextera Mate Pair Sample Preparation Kit, TruSeq PCR-Free Library Prep Kit, and Qiagen QIAseq FX DNA Library kit). All libraries were sequenced with Illumina technology (HiSeq 2500, HiSeq 3000, HiSeq 4000, and NovaSeq 6000) using paired-end settings with read-lengths of 150 bp or 250 bp to generate a minimum of 3,000,000 reads (for individual sample details see Table S5).

Quality filtering, assembly, and bacterial genome binning

Read libraries were trimmed, PhiX contamination filtered, and quality checked using BBMAP v37.61's BBDUK feature (1) and the provided adapter and PhiX databases for contamination detection. All reads below an average q-score of 15 and a minimum length of 100 bp were removed. The minimum contaminant kmer of 21 was used and reads matching the contaminants or below the quality score were filtered out. Libraries were then interleaved (see Jupyter notebook for exact command line).

Individual read libraries were assembled using SPAdes v3.13.1 (2, 3) with the "metagenome" setting and iterative kmers of 21, 31, 41, 51, 61, 71, 81, and 91. The resulting metagenomic assembly scaffolds were then binned using a combination of Anvi'o v6.1 (4, 5) using CONCOCT v1.1.0 (6) and MetaBAT v2.15 (7) (coverage and no coverage options). All contigs shorter than 1000 bp were removed from the assembly prior to mapping and binning. If available, libraries from the same host and location (maximum of three, treated as biological replicates) were used for binning. If three libraries from the same host and location were not available at the time of binning, binning was attempted with libraries from similar hosts or broad scale geographical areas (Dataset S1). Read libraries were mapped to the scaffold assemblies using BBMAP with default settings. The resulting .sam files were converted to .bam files with samtools v1.9 (8) and sorted using the anvi-init-bam script from Anvi'o. Then the default CONCOCT (using v1.1.0) binning workflow within Anvi'o with a minimum length of 1000 bp was used for binning with Anvi'o. The default MetaBAT (v2.15) binning workflow within Anvi'o with a minimum length of 1500 bp (minimum for the program) was also used. A MetaBAT binning attempt with no coverage information was also attempted. All potential bins for each metagenome were then compared using dRep v2.4.2's dereplicate workflow (9) with default settings and the "best" was selected automatically. If no bin was selected, a manual bin selection (using CheckM v1.1.3 (10) with Gammaproteobacteria gene set) and revision of the best MAG (metagenome assembled genomes) using Anvi'o's 'anvi-refine' was attempted as a method of manually improving quality of MAGs. The bins were then checked for completion using CheckM's taxonomy specific workflow using the previously mentioned gene set. MAGs determined to be 90% or more complete and 5% or less contaminated from the gammaproteobacterial gene set in CheckM were used in further downstream analysis without further refinement. MAGs determined to be 90% complete and more than 5% contaminated were manually refined using 'anvi-refine' in an attempt to improve MAG quality. MAGs that were determined to be 90% or more complete and less than 10% contaminated post refinement, referred to as high-quality MAGs, were used in further downstream analysis.

DESMAN was used to calculate the potential number of strains within each MAG. To do so, metagenomic libraries of MAGs with an average coverage of 30 or more were subsampled to 30 coverage by using the "outm" and downsample options in BBMAP using the seed numbers of 1, 11, 42, 47, and 117 to reproduce five subsample readsets. Those under 30 coverage were not subsampled and used as is. To increase the sensitivity, a custom set of 173 single copy genes (Table S8) was created by using the DESMAN specific setting to determine single copy COGs with species groups of interest. These genes were used in place of the original 35 genes in the DESMAN snakemake workflow and the five down sampled replicates were used for each MAG. Potential strain numbers in individual clams/metagenomes were calculated using DESMAN v2.1.1 (11) and the snakemake workflow (12) on the program. The 'best run' of each replicate was taken and considered the number of strains for the metagenome.

Phylogenetic analyses

The taxonomy of all MAGs was checked using GTDB v0.3.3 (13) as a confirmation. Only those MAGs taxonomically assigned to *Sedimenticolaceae* or *Chromatiaceae* were used in this study. All publicly available MAGs of other lucinid symbionts (*Ca.* Thiodiazotropha sp. and *Ca*. Sedimenticola sp.), alongside *Allochromatium vinosum* as an outgroup, were downloaded for use

in this analysis (complete list of accession numbers in Tables S2 and S1). All publicly available MAGs used in this study were quality checked using CheckM's taxonomy specific workflow, which used the previously mentioned Gammaproteobacterial taxonomy set of marker genes. MAGs below 90% completion and/or above 10% contamination were only included in the "Phylogenetic analysis" of this study. Metagenomic read libraries associated with publicly available MAGs (mostly from (14)) were downloaded from NCBI and were also quality filtered using the same workflow as mentioned above. Those metagenomic read libraries that were sequenced to be shorter than 150 bp were filtered using the length cut off of 50 bp.

All available MAGs (irrespective of completion) were used to generate a concatenated gene tree. The CheckM v1.1.3 (10) lineage workflow was used to locate, align, and concatenate the default set of 43 universal marker genes (concatenated marker gene alignment can be found on FigShare (15)). CheckM's automatically generated concatenated protein alignment of these 43 marker genes was then submitted to the W-IQ-TREE server (16) using the default settings (auto substitution model detection, 1000 ultrafast bootstraps, and 1000 replicates of SH-aLRT branch tests). This tree was visualized and rooted with *Allochromatium vinosum* in iTOL (Interactive Tree Of Life) v5 (17). 16S rRNA gene sequences were retrieved from each MAG using BARRNAP v0.9 (18). Near full length 16S rRNA gene sequences (greater than 1200 bp) were aligned using the online SILVA:SINA aligner (19) using default parameters. The alignment was used to generate a phylogeny and visualized using the workflow mentioned previously.

All MAGs were placed into species groups using FastANI v1.3 (20) which was used to determine ANI (average nucleotide identity) values and a program-suggested species cut off of 95% two-way ANI were used. MAGs near the 95% ANI boundary were submitted to jspecies web server (21) to run an ANIb and ANIm test to check the accuracy of species boundaries.

Localization of symbionts in clam gills

The presence of *Ca*. T. taylori within select hosts was visualized with FISH (fluorescence *in situ* hybridization) on 5-µm-thick sections of the gills from *Loripes orbiculatus* (Mauritania, 2018) and *Clathrolucina costata* (Panama, 2019). 16S rRNA gene sequences from *Ca.* T. taylori, assigned to Sedimenticolaceae with SILVA (see Phylogenetic analyses), were used as probe references in probe design. A probe was designed with Geneious Prime (2020.0.5) and Decipher (probe design web tool, (22)). The 16S rRNA gene sequence of *Ca.* T. endolucinida (a co-occurring species of symbiont) was used as a non-target group to prevent false positives during the process. A formamide series from 0-60%, in 10% steps, was carried out to optimize the hybridization conditions for the *Ca*. T. taylori and T. endolucinida probes. A gammaproteobacteria-specific probe (Gam42a-Fluos) was included as a positive control (23). All probe sequences used are available in Table S6.

Gill sections were fixed, post dissection, in a 4% paraformaldehyde, 10% sucrose, pH 7.4, 0.01 M PBS solution overnight at 4°C. The gills were then washed in 10% sucrose, pH 7.4, 0.01 M PBS solution for 10 minutes, three times, followed by an increasing ethanol series of 30%, 50%, and 70% (v/v) for 10 minutes each. Dehydrated gills were stored in 70% ethanol at 4°C until embedding. Gills were embedded in 1% low melting agarose for embedding. Additional dehydration and paraffin wax infiltration was performed using the standard protocol in Automatic Tissue Processor Donatello (Diapath, Italy). For initial infiltration, low melting temperature paraffin was used (ATS-200850, Sanova, Austria). Samples were then embedded in medium melting temperature paraffin (ATS-200856, Sanova, Austria). The paraffin-embedded gills of the clams were cross-sectioned at 5 µm using a Leica microtome (Leica, Germany) and subsequently mounted on SuperfrostPlus adhesion slides (Thermo Scientific, USA) in a 37°C water bath. Sections were then dried in a horizontal position at room temperature overnight. Sections were dewaxed in Roti-Histol (Carl Roth, Germany), 10 minutes for three times, followed by two 10 min washes with absolute ethanol and finalized with three five minute washes of 1X PBS. Dewaxed sections were dried using compressed air and stored at 4°C until use.

Gill sections were hybridized using a hybridization buffer containing 50% Formamide, 0.01% SDS (v/v), 0.1 g/mL Dextran sulfate, 10% blocking reagent (Roche, Switzerland), at a final concentration of 0.9M NaCl, 0.02M Tris-HCl (Table S7). Approximately 15 µl of buffer was used per gill section. 2 µl (10 µmol) of each probe (Table S6) was added to each section. Slides were hybridized for 3 hours at 46°C in dark conditions. To maintain a humid atmosphere within the hybridization chamber during incubation, Kimtech Science precision wipes (Kimberly-Clark, USA) partially soaked in 40% or 50% formamide were located underneath the samples. Following the hybridization, the samples were rinsed with pre-warmed 48°C washing buffer (Table S7), rinsed briefly in Milli-Q water, and rapidly dried using compressed air. Dried slides were stained using 15 µl (1 µg/mL) of DAPI and incubated at room temperature in dark conditions for 15 min. Post incubation, the DAPI was rinsed briefly in 4°C Milli-Q water, three times, and dried. After drying, the sections were mounted using ProLong Glass antifade mounting media (Thermo Fisher Scientific, USA), cured overnight at room temperature and stored at 4°C.

Sections were visualized with a Leica TCS SP8 X confocal laser scanning microscope. Images were taken with a 63X Plan-Apochromat oil immersion objective (Refractive Index of glass slide, immersion oil and antifade mounting medium: 1.52) The Leica software LASX (3.7.2.22383) was used for image acquisition and post-processing if necessary.

Functional annotation and pangenomic analysis of bacterial genomes

Anvi'o's pangenomic workflow was used for orthologous group clustering and functional comparisons. COG (Cluster of Orthologous Groups of proteins) functions were annotated and included using 'anvi-run-ncbi-cogs'. Each MAG's ORF (Open Reading Frame) proteins from Anvi'o were annotated using eggNOG-mapper v2 (24) with eggNOG database v5.0 (25) and these were added to the pangenomic database. All ORF proteins were also used to generate a custom functional annotation set by using all Pfam (protein family) HMMs (26). These ORF proteins were searched for all possible domains using HMMER v3.3 (27) to search against the PfamA reference database v33.1 with the model designated thresholds used as cutoff. This output was then parsed and added as a functional annotation to each MAG. DRAM (Distilled and Refined Annotation of Metabolism) v1.1.1 was also used to annotate and visualize the metabolisms of all high-quality MAGs (28).

All high-quality MAGs in the dataset were also used in an Anvi'o pangenome similar to above with a mcl inflation value of 8. Anvi'o pangenomic genomes database and profiles can be found on Figshare (29, 30). All MAGs were also submitted to the RAST (Rapid Annotation using Subsystem Technology; https://rast.nmpdr.org/) web server for functional annotation using the default RASTtk pipeline (31). Available metagenomic assemblies were screened for genes and proteins of interest using NCBI's blast+ v 2.8.1 (32) with an e-value threshold of $1e^{-16}$ and a word size of 25 (only for blastn). Coverages for genes of interest were also calculated using BBMAP's coverage stats options with a 90% minimum identity to screen for potentially unassembled genes in assemblies.

EggNOG functions and Pfam functions were used for a functional enrichment analysis through 'anvi-get-enriched-functions-per-pan-group'. An enrichment calculation was performed between species of interest using the 95% ANI species cut-offs (as determined by FastANI) as pre-defined groups to create a bacterial interspecies comparison of metabolic functions. Species groups with less than three MAGs were excluded from the enrichment analysis. A second enrichment calculation was performed within species by grouping according to geographical location. Only groups with more than three individuals were used for functional enrichment. Within all analyses, only genes with an 'adjusted q value', a p value adjusted for false discovery rate, of less than 0.05 were considered.

C. orbicularis *metatranscriptome analysis*

Three *C. orbicularis* specimens were collected in Guadeloupe (2016), preserved in RNAlater, and stored at -80°C. Total RNA was extracted using TRIzol (Thermo Fisher Scientific) according to manufacturer's instructions. Ribosomal RNA depletion and library construction were carried out at the Vienna Biocenter Core Facilities GmbH (VBCF, Vienna, Austria) as described in Yuen *et al.* (2019) (33). The RNA-Seq reads were trimmed and processed as previously described by Yuen *et al.* (2019). We used BBMAP with the parameters slow = t, ambiguous = best, minid = 0.99 to align the trimmed and filtered reads to the publicly available MAG of *Ca.* T. endolucinida (GCA_001715975.1), an endosymbiont of *C. orbicularis* from Guadeloupe (1). FeatureCounts was then used to quantify gene level transcript abundances, which were subsequently converted to transcripts per million (34). Metatranscriptomic results can be found in Dataset S7.

Recombination rates and nucleotide diversity of bacterial symbionts

To infer recombination events in bacterial genomes, we used the maximum likelihood implementation of ClonalFrame, ClonalFrameML (35). MAGs (only high quality) were aligned with progressiveMauve, which is part of the Mauve genome alignment package v2.0 (36). Core genes; *i.e*., nucleotide sequences that were shared among all MAGs within a group, were extracted with a custom script, stripSubsetLCBs, which can be accessed through the original publication (37). These core genes were then re-aligned with MUSCLE (38) and cleaned with trimAl (39) with the following parameters: -resoverlap 0.75 -seqoverlap 80. RAxML v8.2.10 (40) was used to build a phylogenetic tree based on this trimmed alignment analogously to (41). All alignments are available on Figshare (42-44). The resulting tree and alignment were fed into ClonalFrameML to calculate the ratio of recombination vs. mutation events with default parameters. All MAGs were used for this analysis (*i.e*., n = 8 for *Ca*. T. weberae, n = 10 for *Ca*. T. lotti, n = 18 for *Ca*. T. endolucinida, and n = 24 for *Ca*. T. taylori). The visualization of the recombination events across the phylogeny was computed in R v6.3.2 with the script cfml_results.R with a few manual modifications, which can be downloaded from the ClonalFrameML Github page (Fig. S1A-C). One hundred simulations were run to calculate confidence intervals of the estimates. The cleaned alignments were also used to (i) estimate average nucleotide diversities inside and outside the clonal frame with GUBBINS (Genealogies Unbiased By recomBinations In Nucleotide Sequences (45)), and (ii) build a phylogenetic tree of all MAGs within the *Ca*. T. taylori group (n = 24) using only the clonal frame. For the latter, the alignment was again submitted to the W-IQ-TREE server using the default settings (auto substitution model detection, 1000 ultrafast bootstraps, and 1000 replicates of SH-aLRT branch tests). This tree was visualized and rooted with *Ca.* T. sp. “RUGA” in iTOL. All scripts used for the analysis can be found in the associated Jupyter Notebook (46).

Data and analysis scripts deposition

NCBI Bioproject ID: PRJNA679177

Biosample Accession Numbers: SAMN16825223 - SAMN16825285 for MAGs and SAMN16952162 - SAMN16952207 for the corresponding raw read sets.

FigShare: https://figshare.com/projects/A_globally_distributed_symbiont_challenges_host_specificity_in_lucinid_clams/93398

Scripts: https://doi.org/10.6084/m9.figshare.13295912.v5

SI Results

Storage Compounds

All species are also predicted to be able to store and utilize glycogen and polyhydroxyalkanoates, as indicated by RAST annotations and the presence of genes annotated as Poly(3-hydroxyalkanoate) polymerase (phaC).

Phosphorus

All species have genes for phosphate ABC transporter proteins (pstSCAB genes and phoU), which suggest the ability to take up extracellular phosphate for use inside the cell. All species also have the genes for synthesizing and utilizing polyphosphate as a storage compound for excess phosphate/energy. Notably *Ca.* T. weberae and *Ca.* T. sp. RUGA encoded for an organophosphonate utilization operon (phnACDEFGHIJKMNOP genes), which suggest the capability of these two species to utilize compounds containing C-P bonds (47). These compounds (phosphonates) are found to be up to 10% of the total bioavailable phosphorus in marine environments.

Supplementary Discussion

Naming conventions for Lucinid symbionts should be altered to avoid host-specific nomenclature

There are now two lucinid symbionts that have been found to associate with multiple host species (*Ca*. T. taylori and *Ca*. T. endolucinida) and several host species that associate with different symbiont species (four symbiont species within *Ctena orbiculata* and three symbiont species with *Loripes orbiculatus*). With only a small fraction of host species sampled so far, it is likely that there will be more symbionts that associate with multiple host species. This causes host-centric names like "*Ca*. T. endoloripes", "*Ca*. T. endolucinida", and "*Ca*. Sedimenticola endophacoides" to potentially be invalidated and less insightful with future discoveries. Previously, based on limited sampling, it had been assumed that these symbionts only occurred within single host species. While the species name "*Ca*. T. endolucinida" is accurate as it is found in many lucinid hosts, it is not the only species that is found in many hosts, just as "*Ca.* S. endophacoides" might not be the only host within Phacoides. A host centric naming system also creates issues for any potential *Ca*. Thiodiazotropha that are first found in the environment, as found recently in seagrass using FISH (fluorescent *in situ* hybridization). In this study, we propose a shift in the naming of these species away from a host centric system to a more applicable system of naming focused on those that have made contributions to the fields of lucinid taxonomy and chemosymbiosis. Within the umbrella of "*Ca*. T. endoloripes" species, previously based on 16S rRNA gene sequences, we found metagenome-assembled genomes (MAGs) that grouped into two different species. Note that we identified two specimens of *Loripes orbiculatus* hosting both symbionts living peacefully in the same gill. We are proposing the names "*Ca.* T. weberae" and "*Ca*. T. lotti" after Miriam Weber and Christian Lott, whose work in the bay of Fetovaia on Elba Island, Italy led to the discovery of a *Loripes orbiculatus* population that kick-started the research on this species and its symbionts. The cosmopolitan symbiont associated with eight host species across three oceans will be named "*Ca* T. taylori" after Dr. John Taylor. Dr. John Taylor has spent decades studying and describing the biodiversity within the Lucinidae. His invaluable work on lucinid taxonomy, morphology and systematics has built the foundations for many others to pursue careers studying these wonderful organisms. Our work was only possible because of his contributions, discussions, and extensive museum collection.

Figure S1: Corrected *Ca*. Thiodiazotropha phylogenies using ClonalFrameML

A)

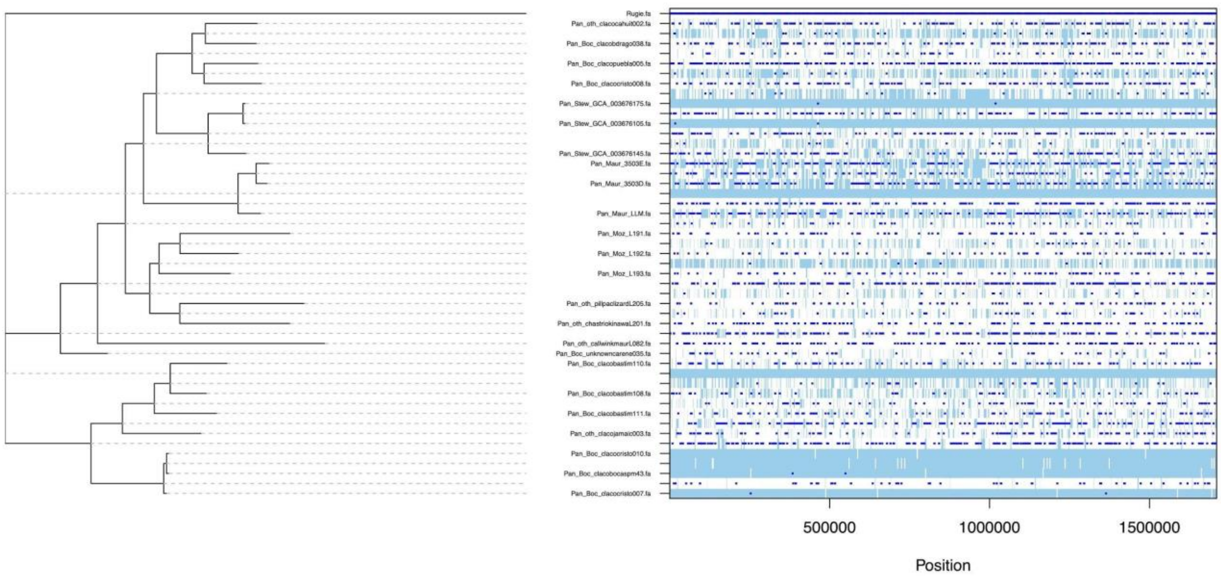

B)

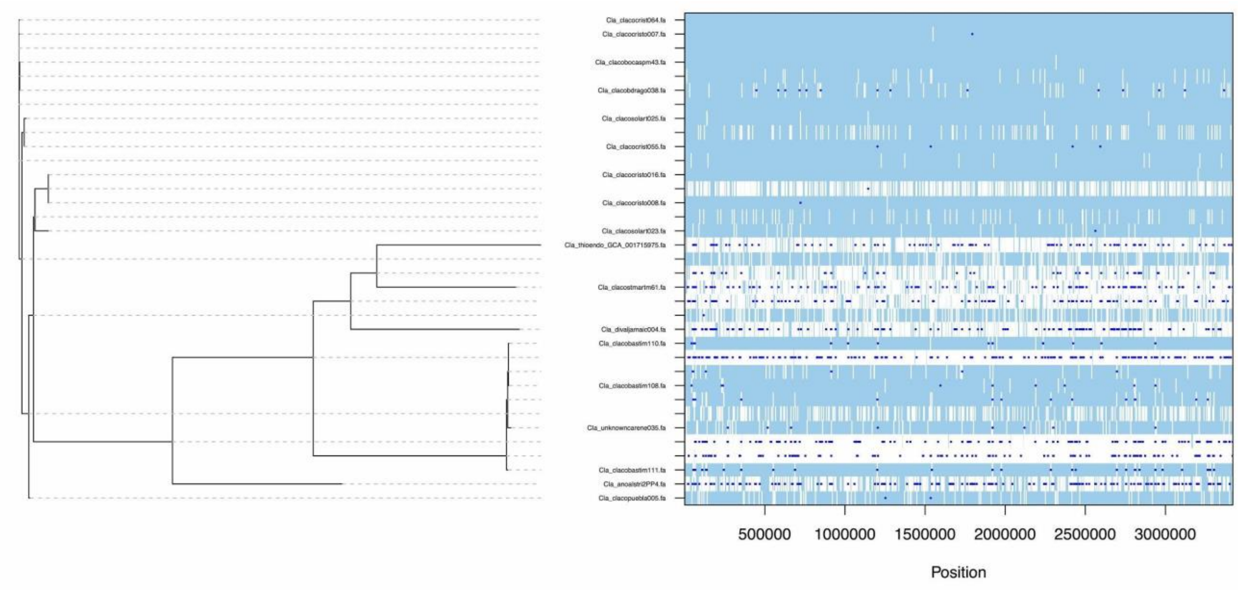

C)

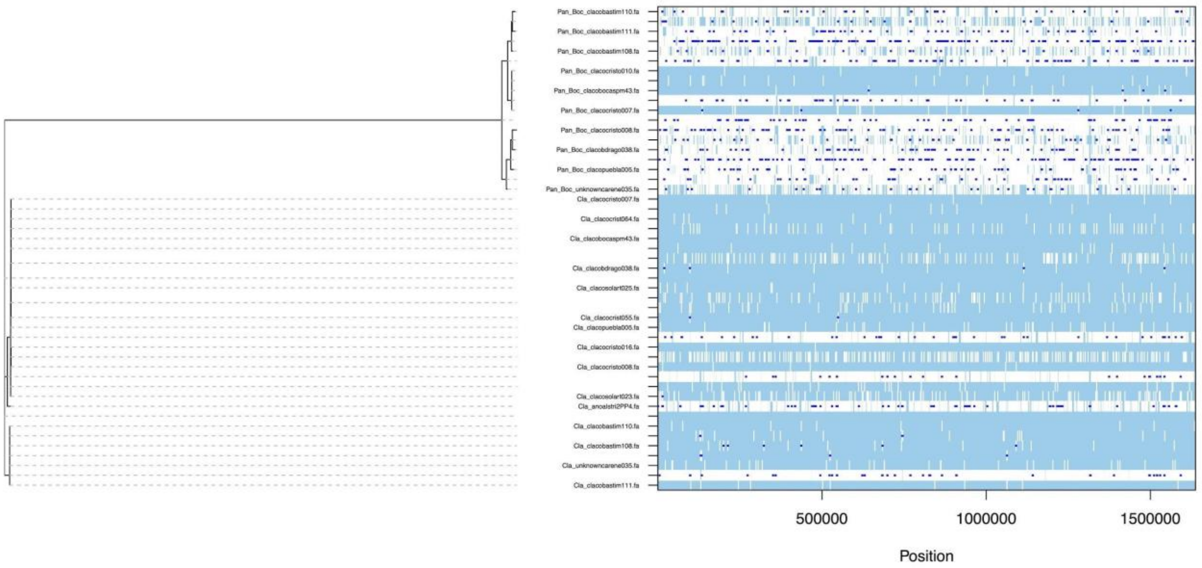

D)

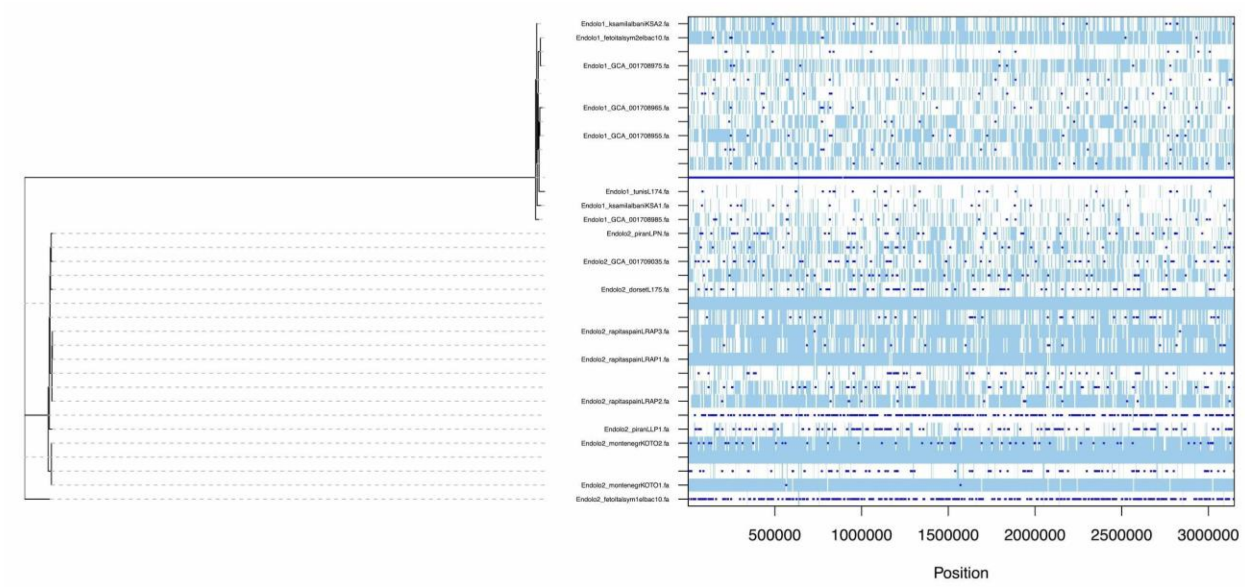

Figure S1: ClonalFrameML recombination corrected phylogenies. Reconstructed substitutions (white vertical bars) are shown for each branch of the maximum likelihood (ML) tree. The ML tree was constructed within ClonalFrameML and is based on the non-recombining part of the genome (see Material and Methods section and Figure 2 in the main manuscript). Briefly, metagenomic-assembled genomes (MAGs) were aligned using ProgressiveMauve and the shared genes in this alignment were extracted and concatenated (x-axis on heatmap, showing position of this concatenated alignment). Dark blue horizontal bars indicate recombination events detected by the analysis. Light blue horizontal bars indicate no change between two branches. MAG labels correspond to names used in the bioinformatic pipeline described in the associated Jupyter Notebook and labels on GenBank. A) *Ca*. T. taylori and *Ca*. T. RUGA, B) *Ca*. T. endolucinida, C) *Ca*. T. taylori and *Ca*. T. endolucinida combined, and D) *Ca*. T. weberae and lotti combined.

Figure S2: Summary of predicted symbiont metabolism based on assembled gene sequences

A)

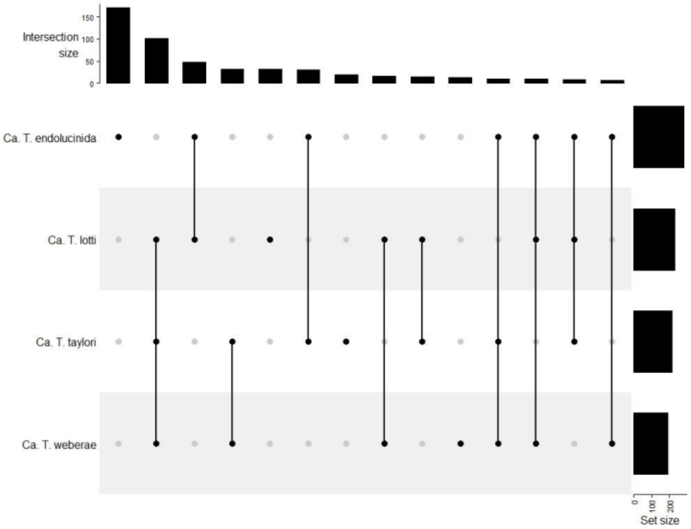

B)

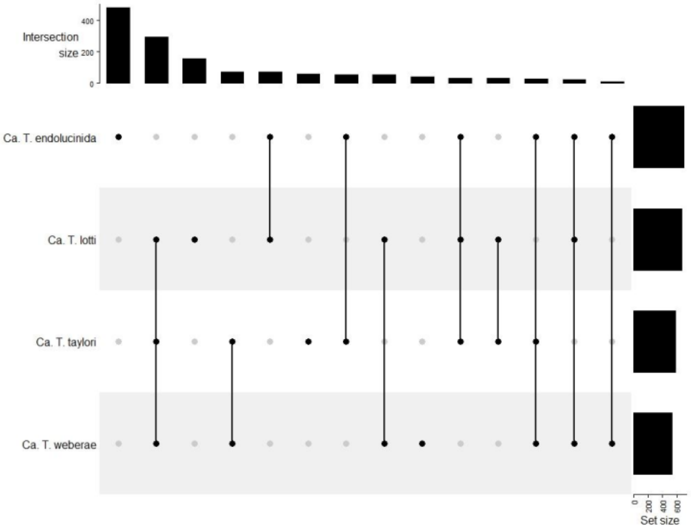

Figure S2: A) Upset plot displaying the significantly enriched Pfams (protein families) between the four species of interest (*Ca.* T. endolucinida, taylori, weberae, and lotti). B) Upset plot displaying the significantly enriched functions of "EGGNOG_BACT" (a combination of eggNOG annotation gene name and free text) between the four species of interest. *Ca. Thiodiazotropha endolucinida* has the most functions labeled as enriched while the node of *Ca.* T. taylori*, Ca.* T. weberae, and *Ca.* T. lotti had the second most, likely due to the phylogenetic relationship. Intersect size represents the number of enriched groups contained within all species in the group (indicated with black dots in the column). Set size represents the total number of enriched groups within individual species.

Figure S3: 16S rRNA gene tree of lucinid symbionts

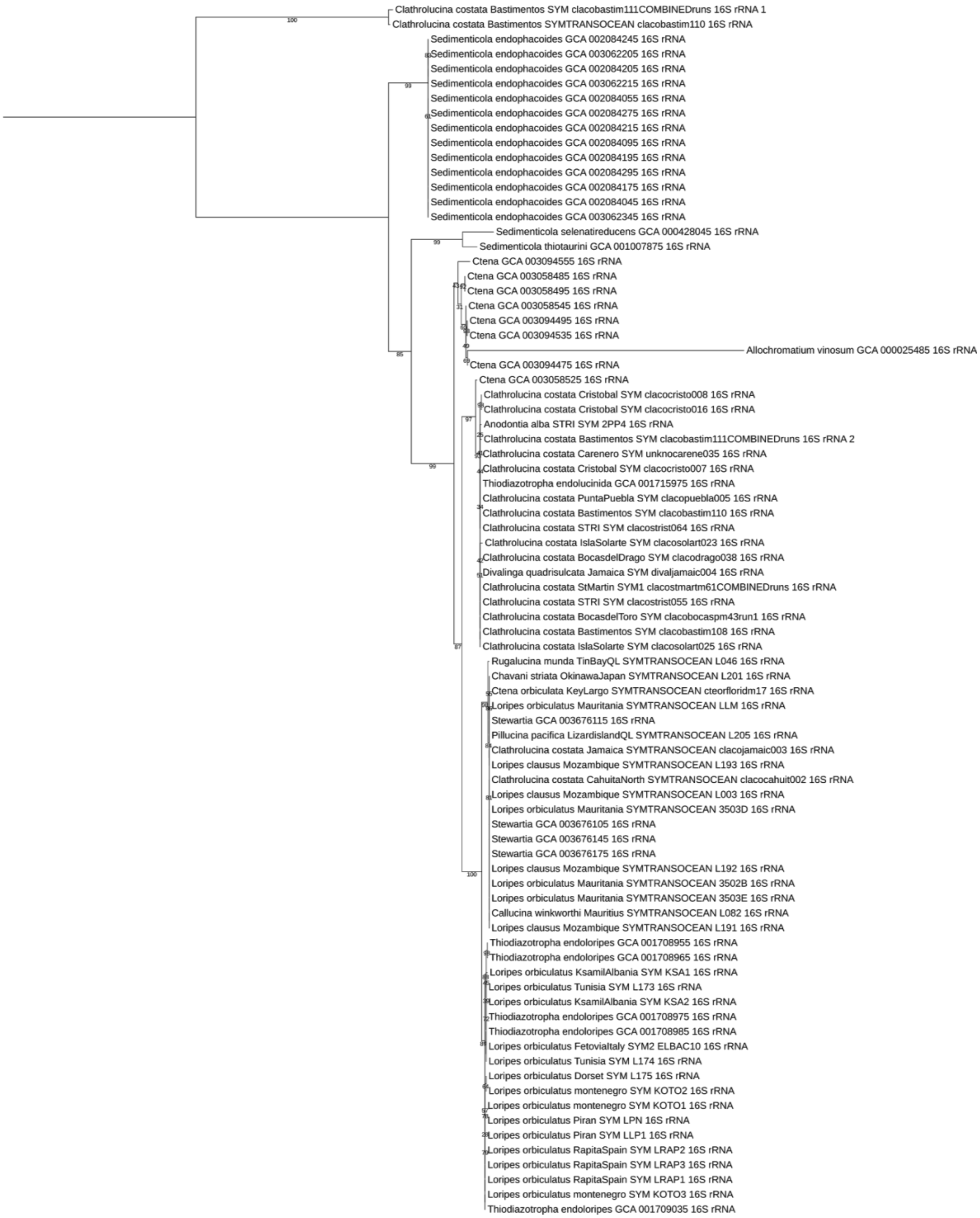

Figure S3: Phylogenetic relationship of lucinid endosymbionts based on full-length 16S rRNA gene sequences. Tree is rooted on contaminant rRNA sequences that were identified in clam gills as non-symbiont sequences (*Endozoicomonas sp.*). The substitution model K2P+I+G4 was automatically chosen by IQtree.

Table S1. DESMAN strain diversity estimates of high-quality MAGs

MAG	Max Strains	Trusted Strains	Species
Anodontia_alba_STRI_SYM_2PP4	2	1	*Ca*. T. endolucinida
Callucina_winkworthi_Mauritius_SYMTRANSOCEAN_L082	1	1	*Ca*. T. taylori
Chavani_striata_OkinawaJapan_SYMTRANSOCEAN_L201	2	2	*Ca*. T. taylori
Clathrolucina_costata_Bastimentos_SYM_clacobastim108	1	1	*Ca*. T. endolucinida
Clathrolucina_costata_Bastimentos_SYM_clacobastim110	2	2	*Ca*. T. endolucinida
Clathrolucina_costata_Bastimentos_SYM_clacobastim111COMBINEDruns	2	2	*Ca*. T. endolucinida
Clathrolucina_costata_Bastimentos_SYMTRANSOCEAN_clacobastim108	1	1	*Ca*. T. taylori
Clathrolucina_costata_Bastimentos_SYMTRANSOCEAN_clacobastim110	1	1	*Ca*. T. taylori
Clathrolucina_costata_Bastimentos_SYMTRANSOCEAN_clacobastim111COMBINEDruns	1	1	*Ca*. T. taylori
Clathrolucina_costata_BocasdelDrago_SYM_clacodrago038	2	2	*Ca*. T. endolucinida
Clathrolucina_costata_BocasdelDrago_SYMTRANSOCEAN_clacodrago038	1	1	*Ca*. T. taylori
Clathrolucina_costata_BocasdelToro_SYM_clacobocaspm43run1	2	2	*Ca*. T. endolucinida
Clathrolucina_costata_BocasdelToro_SYMTRANSOCEAN_clacobocaspm43run1	1	1	*Ca*. T. taylori
Clathrolucina_costata_CahuitaNorth_SYMTRANSOCEAN_clacocahuit002	3	1	*Ca*. T. taylori
Clathrolucina_costata_Carenero_SYMTRANSOCEAN_unknocarene035	1	1	*Ca*. T. taylori
Clathrolucina_costata_Carenero_SYM_unknocarene035	2	2	*Ca*. T. endolucinida
Clathrolucina_costata_Cristobal_SYM_clacocristo007	2	2	*Ca*. T. endolucinida
Clathrolucina_costata_Cristobal_SYM_clacocristo008	3	2	*Ca*. T. endolucinida
Clathrolucina_costata_Cristobal_SYM_clacocristo016	3	2	*Ca*. T. endolucinida
Clathrolucina_costata_Cristobal_SYMTRANSOCEAN_clacocristo007	1	1	*Ca*. T. taylori
Clathrolucina_costata_Cristobal_SYMTRANSOCEAN_clacocristo008	1	1	*Ca*. T. taylori
Clathrolucina_costata_Cristobal_SYMTRANSOCEAN_clacocristo010	1	1	*Ca*. T. taylori
Clathrolucina_costata_IslaSolarte_SYM_clacosolart023	3	2	*Ca*. T. endolucinida
Clathrolucina_costata_IslaSolarte_SYM_clacosolart025	2	2	*Ca*. T. endolucinida
Clathrolucina_costata_Jamaica_SYMTRANSOCEAN_clacojamaic003	2	2	*Ca*. T. taylori
Clathrolucina_costata_PuntaPuebla_SYM_clacopuebla005	3	1	*Ca*. T. endolucinida
Clathrolucina_costata_PuntaPuebla_SYMTRANSOCEAN_clacopuebla005	1	1	*Ca*. T. taylori
Clathrolucina_costata_StMartin_SYM1_clacostmartm61COMBINEDruns	1	1	*Ca*. T. endolucinida
Clathrolucina_costata_STRI_SYM_clacostrist055	1	1	*Ca*. T. endolucinida
Clathrolucina_costata_STRI_SYM_clacostrist064	2	2	*Ca*. T. endolucinida
Divalinga_quadrisulcata_Jamaica_SYM_divaljamaic004	3	2	*Ca*. T. endolucinida
Loripes_clausus_Mozambique_SYMTRANSOCEAN_L191	2	2	*Ca*. T. taylori
Loripes_clausus_Mozambique_SYMTRANSOCEAN_L192	1	1	*Ca*. T. taylori
Loripes_clausus_Mozambique_SYMTRANSOCEAN_L193	1	1	*Ca*. T. taylori
Loripes_orbiculatus_Dorset_SYM_L175	2	2	*Ca*. T. lotti
Loripes_orbiculatus_FetoviaItaly_SYM1_ELBAC10	3	1	*Ca*. T. lotti
Loripes_orbiculatus_FetoviaItaly_SYM2_ELBAC10	1	1	*Ca*. T. weberae
Loripes_orbiculatus_KsamilAlbania_SYM_KSA1	1	1	*Ca*. T. weberae
Loripes_orbiculatus_KsamilAlbania_SYM_KSA2	1	1	Ca. T. weberae
Loripes_orbiculatus_Mauritania_SYMTRANSOCEAN_3503D	1	1	*Ca*. T. taylori
Loripes_orbiculatus_Mauritania_SYMTRANSOCEAN_3503E	1	1	*Ca*. T. taylori
Loripes_orbiculatus_Mauritania_SYMTRANSOCEAN_LLM	1	1	*Ca*. T. taylori
Loripes_orbiculatus_montenegro_SYM_KOTO1	2	2	*Ca*. T. lotti

Loripes_orbiculatus_montenegro_SYM_KOTO2	1	1	*Ca*. T. lotti
Loripes_orbiculatus_Piran_SYM_LLP1	2	2	*Ca*. T. lotti
Loripes_orbiculatus_Piran_SYM_LPN	2	1	*Ca*. T. lotti
Loripes_orbiculatus_RapitaSpain_SYM_LRAP1	1	1	*Ca*. T. lotti
Loripes_orbiculatus_RapitaSpain_SYM_LRAP2	2	1	*Ca*. T. lotti
Loripes_orbiculatus_RapitaSpain_SYM_LRAP3	1	1	*Ca*. T. lotti
Loripes_orbiculatus_Tunisia_SYM_L173	2	2	*Ca*. T. weberae
Loripes_orbiculatus_Tunisia_SYM_L174	2	2	*Ca*. T. weberae
Pillucina_pacifica_LizardislandQL_SYMTRANSOCEAN_L205	2	2	*Ca*. T. taylori
Stewartia_GCA_003676105	2	2	*Ca*. T. taylori
Stewartia_GCA_003676145	2	1	*Ca*. T. taylori
Stewartia_GCA_003676175	2	2	*Ca*. T. taylori
Thiodiazotropha_endoloripes_GCA_001708955	1	1	*Ca*. T. weberae
Thiodiazotropha_endoloripes_GCA_001708965	2	2	*Ca*. T. weberae
Thiodiazotropha_endoloripes_GCA_001708975	2	2	*Ca*. T. weberae
Thiodiazotropha_endoloripes_GCA_001708985	2	2	*Ca*. T. weberae
Thiodiazotropha_endoloripes_GCA_001709035	3	2	*Ca*. T. lotti

Table S2. Summary of metabolisms of primary species groups of interest

	Ca. T. taylori	*Ca*. T. endolucinida	*Ca*. T. weberae	*Ca*. T. lotti	*Ca*. T. RUGA
**Carbon metabolism**					
CBB cycle, form I (RuBisCO)	+	+	+	+	+
CBB cycle, form II (RuBisCO)	-	+	-	-	-
Glycolysis	+	+	+	+	+
Oxidative TCA cycle	+	+	+	+	+
Glyoxylate shunt	+	+	+	+	+
Methylotrophy pathway (pqq)	+	+	-	-	+
Pyruvate phosphate dikinase	+	+	+	+	+
PEP synthase	+	+	+	+	+
Pyruvate synthase (PFOR)	+	+	-	-	+
Pyruvate carboxylase	-	+	-	-	-
PEP carboxylase	+	+	+	+	+
PEP carboxylase (GTP)	+	+	+	+	+
**Nitrogen metabolism**					
Diazotrophy, nitrogenase	+	+	+	+	+
Respiratory nitrate reductase	-	+	-	-	-
Copper-containing nitrite reductase (NO-forming)	- *	+	-	+	+
Nitric-oxide reductase	+	+	+	+	+
Nitrous-oxide reductase	+	+	+	+	+
Periplasmic nitrate reductase	+	+	+	+	+
Nitrite reductase NADPH subunit	+	+	+	+	+
Urease	+	-	+	-	-
Urea amidolyase	+	-	+	+	+
Ammonia assimilation	+	+	+	+	+
Ammonia assimilation	+	+	+	+	+
**Sulfur metabolism**					
Sulfide-quinone reductase	+	+	+	+	+
Sulfur oxidation pathway	+	+	+	+	+
Dsr	+	+	+	+	+
DsrMKJOP complex	+	+	+	+	+
Apr	+	+	+	+	+
Flavocytochrome c sulfide dehydrogenase	+	+	+	+	+
**Phosporous metabolism**					
Phosphate transport system	+	+	+	+	+
Polyphosphate synthesis	+	+	+	+	+

Organophosphonate utilization	-	-	+	-	+
**Iron metabolism**					
Iron (+2) transport	+	+	+	+	+
**Secretion systems**					
Type I	+	+	+	+	+
Type IV	+	+	+	+	+
Type VI	+	+	+	+	+
**Hydrogenases**					
Hydrogenase-1	+	+	+	+	+
Hydrogenase-2	-*	+	-	+	+
Uptake hydrogenase	+	+	+	+	+
NAD-reducing hydrogenase	+	+	+	+	+
**Energy**					
Rnf transporter	+	+	+	+	+
V-type ATPase	+	+	+	+	+
Cyt c oxidase cbb3 type	+	+	+	+	+
cytochrome c2	-*	+	+	+	+
c553	+	+	+	+	+
c551/c552	+	+	+	+	+
FX	+	+	+	+	+
C4	+	+	+	+	+
**Motility**					
Flagella	+	+	+	+	+
**Storage compounds**					
Glycogen	+	+	+	+	+
Polyhydroxyalkanoates	+	+	+	+	+
**MIscellanous**					
Kai (circadian clock)	+	-	-	-	-

*These genes were only present in the high-quality MAGs of *Ca*. T. taylori associated with *Stewartia floridana*, from Florida, USA.

Table S3. Total enriched functions by species group

Ca. T endolucinida	*Ca*. T. taylori	*Ca*. T. weberae	*Ca*. T. lotti	# of Enriched Pfam Functions	# of Enriched eggNOG Functions
X	X	X		10	29
X	X		X	9	32
X	X			30	57
X		X	X	10	25
X		X		7	10
X			X	48	72
X				170	478
	X	X	X	102	296
	X	X		32	74
	X		X	15	31
	X			19	61
		X	X	17	56
		X		13	40
			X	32	159
total enriched				514	1420
total functions				2361	4401
% of total found to be enriched				21.77043626	32.26539423

Table S4. Recombination statistics per species group, calculated by ClonalFrameML

Species	Length of genome alignment (bp)	Recombination events	Average length of events (bp)	Recombination to mutation ratio (95% C.I.)
Ca. T. taylori	1,751,700	103	596	0.814 (0.809 - 0.819)
Florida MAGs	1,751,700	47	632	0.859 (0.692-0.955)
Mauritania MAGs	1,751,700	167	533	1.521 (1.277-1.765)
Panama MAGs	1,751,700	99	431	1.033 (0.963-1.103)
Mozambique MAGs	1,751,700	84	323	0.938 (0.878-0.997)
Ca. T. weberae	3,149,280	31	190	0.043 (0.041-0.046)
Ca. T. lotti	3,149,280	189	465	0.085 (0.083-0.087)
Ca. T. endolucinida	3,420,600	41	385	0.082 (0.079-0.084)
Co-occuring Pair				
Ca. T. taylori - *Ca.* T. endolucinida	1,664,616	15	662	0.0066 (0.0063-0.0069)
Ca. T. weberae - *Ca.* T. lotti	3,149,280	18	433	0.053 (0.0522-0.0541)

Table S5. Sequencing methods used in this manuscript

Method # (Metadata Table)	Extraction Kit	Incubation Time in Proteinase K	QC Method	RNAase Treatment	Library prep kit	Sequencer	Sequencing Facility
1	Analytikajena Innuprep DNA Mini Kit	incubation 2-10 hours	Agilent 4200 TapeStation System	NO	NEBNext® Ultra™ II FS DNA Library Prep Kit for Illumina	Illumina NovaSeq 6000 System, SP flowcell (2x150 bp)	JMF
2	Qiagen Blood and Tissue Kit	incubation 20 hours	Qubit dsDNA HS Assay Kit	YES	Qiagen QIAseq FX DNA Library kit	Illumina HiSeq 4000 paired-end	UC-DAVIS
3	Qiagen Blood and Tissue Kit	incubation 3-10 hours	Agilent 4200 TapeStation System	NO	TruSeq PCR-Free Library Prep Kit	Illumina HiSeq 3000/4000 System, paired-end (2x150 bp)	BSF
4	Qiagen Blood and Tissue Kit	incubation 3-10 hours	Agilent 4200 TapeStation System	NO	Nextera Mate Pair Sample Preparation Kit	Illumina HiSeq2500 system, paired end reads (2x250bp)	MPI
5	Qiagen Blood and Tissue Kit	incubation 3-10 hours	Agilent 4200 TapeStation System	NO	NEBNext Ultra DNA v2 Library Prep Kit	Illumina HiSeq3000 system, paired end reads (2x150bp)	MPI
6	Analytik Jena Innuprep DNA Mini Kit	incubation 2-10 hours	Agilent 4200 TapeStation System	NO	NEBNext® Ultra™ II FS DNA Library Prep Kit for Illumina	Illumina HiSeq 3000/4000 System, paired-end (2x150 bp)	BSF

Table S6. FISH probes used in this manuscript

Probe	Target species	Sequence (5'-3')	Dye used	Formamide % used
Trans1246*	*Ca*. T. taylori	CGGGTTCGCGGCTCTCT	Cy3	50%
endolucinida127*	*Ca*. T. endolucinida	CCCACTACTAGGCAGATACCT	Cy5	50%
Gam42a	Gammaproteobacteria	GCC TTT CCA CAT GGT TT	Cy5	50%
EUBI-III	Bacteria	Mixed	Cy3	50%
EUK516	Eukaryote	ACC AGA CTT GCC CTC C	Fluos	50%

*Lucinid gills are low diversity systems that have few symbionts. This low diversity system enabled the creation of FISH probes that would target specific symbionts within individual hosts. Probes for the co-occurring symbionts found within the Caribbean were created using the minimal mismatches of the 16S rRNA gene, as the 16S rRNA gene sequences of these symbionts are 97.5% identical. A probe targeting *Ca*. T. taylori was created, called trans1246, that aligned near the 1246th nucleotide of the 16S rRNA gene. This probe was a perfect match to the *Ca*. T. taylori 16S rRNA gene and contained three mismatches to Ca. T. endolucinida. A probe targeting Ca. T. endolucinida was created, called endolucinda127, that aligned near the 127th nucleotide of the 16S rRNA gene. This probe was a perfect match to the *Ca*. T. endolucinida 16S rRNA gene and contained a single mismatch to *Ca*. T. taylori. The binding and specificity of these probes was tested through a formamide curve. It was determined that both probes bound successfully at 50% formamide with no non-specific binding to the other symbiont.

Table S7. FISH reagents

Reagent	CARD FISH Buffer (6 mL) 40% FA	CARD FISH Buffer (6 mL) 50% FA	Wash Buffer (50 mL) 50% FA
5M NaCl	1.08	1.08	0.18
1M Tris/HCl	0.12	0.12	1
Milli Q	2.4	1.8	48.3
Dextran sulfate	0.6 g	0.6 g	-
Formamide	2.4	3	-
Blocking reagent	0.6	0.6	-
10% SDS	6 µL	6 µL	50 µL
0.5M EDTA	-	-	0.5

Table S8. List of the COG HMMs used in the DESMAN analysis

COG0005	COG0245	COG0774	COG1722	COG2975
COG0006	COG0248	COG0777	COG1758	COG2983
COG0012	COG0253	COG0779	COG1762	COG3016
COG0013	COG0271	COG0794	COG1826	COG3026
COG0016	COG0285	COG0805	COG1862	COG3071
COG0018	COG0287	COG0812	COG1872	COG3073
COG0026	COG0315	COG0815	COG1883	COG3087
COG0031	COG0322	COG0816	COG1897	COG3100
COG0041	COG0325	COG0820	COG1910	COG3104
COG0044	COG0327	COG0837	COG1923	COG3107
COG0082	COG0331	COG0858	COG1932	COG3116
COG0101	COG0337	COG1044	COG1952	COG3143
COG0106	COG0344	COG1156	COG2001	COG3150
COG0107	COG0350	COG1218	COG2127	COG3159
COG0118	COG0354	COG1250	COG2153	COG3202
COG0124	COG0357	COG1314	COG2166	COG3235
COG0125	COG0361	COG1381	COG2201	COG3265
COG0127	COG0455	COG1386	COG2264	COG3279
COG0128	COG0459	COG1388	COG2331	COG3518
COG0133	COG0468	COG1391	COG2350	COG3521
COG0135	COG0496	COG1394	COG2363	COG3705
COG0136	COG0536	COG1409	COG2834	COG3725
COG0138	COG0541	COG1426	COG2840	COG3764
COG0139	COG0544	COG1489	COG2847	COG3842
COG0140	COG0602	COG1514	COG2862	COG3913
COG0143	COG0603	COG1526	COG2884	COG4103
COG0149	COG0613	COG1527	COG2891	COG4147
COG0164	COG0656	COG1539	COG2901	COG4795
COG0173	COG0669	COG1544	COG2915	COG5405
COG0177	COG0688	COG1559	COG2919	
COG0182	COG0689	COG1576	COG2921	
COG0193	COG0696	COG1587	COG2922	
COG0195	COG0706	COG1592	COG2938	
COG0215	COG0707	COG1666	COG2941	
COG0234	COG0733	COG1686	COG2967	
COG0236	COG0757	COG1705	COG2972	

Dataset S1 (separate file).

Sample metadata for individual metagenomes and MAGs.

Dataset S2 (separate file).

Average nucleotide identity values, calculated through FastANI and Jspecies, grouped by species.

Dataset S3 (separate file).

Presence of genes of interest in all high-quality MAGs, using eggNOG and RAST as primary sources of functional information.

Dataset S4 (separate file).

Enriched metabolic functions by species group, assessed using eggNOG and PFAM as functional annotation sources.

Dataset S5 (separate file).

Absence of genes of interest confirmation using BLAST, annotation, and coverage.

Dataset S6 (separate file).

GUBBINS values for primary species of interest.

Dataset S7 (separate file).

Annotated RNA-seq counts from *C. orbicularis* gill sample.

SI References

1. B. Bushnell (2014) BBMap: a fast, accurate, splice-aware aligner. (Lawrence Berkeley National Lab, LBNL), Berkeley, CA, United States.
2. S. Nurk, D. Meleshko, A. Korobeynikov, P. A. Pevzner, metaSPAdes: a new versatile metagenomic assembler. *Genome Res.* **27**, 824-834 (2017).
3. S. Nurk *et al.* (2013) Assembling genomes and mini-metagenomes from highly chimeric reads. in *Research in Computational Molecular Biology* (Springer Berlin Heidelberg), pp 158-170.
4. A. Murat Eren *et al.*, Anvi'o: an advanced analysis and visualization platform for 'omics data. *PeerJ* **3**, e1319 (2015).
5. T. O. Delmont, A. M. Eren, Linking pangenomes and metagenomes: the *Prochlorococcus* metapangenome. *PeerJ* **6**, e4320 (2018).
6. J. Alneberg *et al.*, Binning metagenomic contigs by coverage and composition. *Nat. Methods* **11**, 1144-1146 (2014).
7. D. D. Kang *et al.*, MetaBAT 2: an adaptive binning algorithm for robust and efficient genome reconstruction from metagenome assemblies. *PeerJ* **7**, e7359 (2019).
8. H. Li *et al.*, Genome Project Data Processing Subgroup. 2009. The Sequence alignment/map (SAM) format and SAMtools. *Bioinformatics* **1000**, 2078-2079.
9. M. R. Olm, C. T. Brown, B. Brooks, J. F. Banfield, dRep: a tool for fast and accurate genomic comparisons that enables improved genome recovery from metagenomes through de-replication. *ISME J.* **11**, 2864-2868 (2017).
10. D. H. Parks, M. Imelfort, C. T. Skennerton, P. Hugenholtz, G. W. Tyson, CheckM: assessing the quality of microbial genomes recovered from isolates, single cells, and metagenomes. *Genome Res.* **25**, 1043-1055 (2015).
11. C. Quince *et al.*, DESMAN: a new tool for de novo extraction of strains from metagenomes. *Genome Biol.* **18**, 181 (2017).
12. J. Köster, S. Rahmann, Snakemake-a scalable bioinformatics workflow engine. *Bioinformatics* **28**, 2520-2522 (2012).
13. P.-A. Chaumeil, A. J. Mussig, P. Hugenholtz, D. H. Parks, GTDB-Tk: a toolkit to classify genomes with the Genome Taxonomy Database. *Bioinformatics* **36**, 1926-1927 (2019).
14. S. J. Lim *et al.*, Extensive thioautotrophic gill endosymbiont diversity within a single *Ctena orbiculata* (Bivalvia: Lucinidae) population and implications for defining host-symbiont specificity and species recognition. *mSystems* **4**, e00280-19 (2019).
15. J. T. Osvatic, L. G. E. Wilkins, Marker gene alignment for phylogenetic symbiont tree. FigShare. http://dx.doi.org/10.6084/m9.figshare.13326038.v1. Deposited 2020/12/3.
16. J. Trifinopoulos, L.-T. Nguyen, A. von Haeseler, B. Q. Minh, W-IQ-TREE: a fast online phylogenetic tool for maximum likelihood analysis. *Nucleic Acids Res.* **44**, 232-235 (2016).
17. I. Letunic, P. Bork, Interactive Tree Of Life (iTOL) v4: recent updates and new developments. *Nucleic Acids Res.* **47**, 256-259 (2019).
18. T. Seemann (2013) barrnap 0.9 : rapid ribosomal RNA prediction.
19. E. Pruesse, J. Peplies, F. O. Glöckner, SINA: accurate high-throughput multiple sequence alignment of ribosomal RNA genes. *Bioinformatics* **28**, 1823-1829 (2012).
20. C. Jain, L. M. Rodriguez-R, A. M. Phillippy, K. T. Konstantinidis, S. Aluru, High throughput ANI analysis of 90K prokaryotic genomes reveals clear species boundaries. *Nat. Commun.* **9**, 5114 (2018).
21. M. Richter, R. Rosselló-Móra, F. Oliver Glöckner, J. Peplies, JSpeciesWS: a web server for prokaryotic species circumscription based on pairwise genome comparison. *Bioinformatics* **32**, 929-931 (2016).
22. E. S. Wright, L. S. Yilmaz, A. M. Corcoran, H. E. Ökten, D. R. Noguera, Automated design of probes for rRNA-targeted fluorescence in situ hybridization reveals the advantages of using dual probes for accurate identification. *Appl. Environ. Microbiol.* **80**, 5124-5133 (2014).

23. W. Manz, R. Amann, W. Ludwig, M. Wagner, K.-H. Schleifer, Phylogenetic oligodeoxynucleotide probes for the major subclasses of proteobacteria: problems and solutions. *Syst. Appl. Microbiol.* **15**, 593-600 (1992).
24. J. Huerta-Cepas *et al.*, Fast genome-wide functional annotation through orthology assignment by eggNOG-Mapper. *Mol. Biol. Evol.* **34**, 2115-2122 (2017).
25. J. Huerta-Cepas *et al.*, eggNOG 5.0: a hierarchical, functionally and phylogenetically annotated orthology resource based on 5090 organisms and 2502 viruses. *Nucleic Acids Res.* **47**, 309-314 (2019).
26. S. El-Gebali *et al.*, The Pfam protein families database in 2019. *Nucleic Acids Res.* **47**, 427-432 (2019).
27. Howard Hughes Medical (2011) hmmscan :: search sequence(s) against a profile database, http://hmmer.org.
28. M. Shaffer *et al.*, DRAM for distilling microbial metabolism to automate the curation of microbiome function. *Nucleic Acids Res.* **48**, 8883-8900 (2020).
29. J. T. Osvatic, L. G. E. Wilkins, Pangenome genomes database for Anvi'o. FigShare. http://dx.doi.org/10.6084/m9.figshare.13325744.v1. Deposited 2020/12/3.
30. J. T. Osvatic, L. G. E. Wilkins, Pangenome profiles database for Anvi'o. FigShare. http://dx.doi.org/10.6084/m9.figshare.13325723.v2. Deposited 2020/12/3.
31. T. Brettin *et al.*, RASTtk: a modular and extensible implementation of the RAST algorithm for building custom annotation pipelines and annotating batches of genomes. *Sci. Rep.* **5**, 8365 (2015).
32. C. Camacho *et al.*, BLAST+: architecture and applications. *BMC Bioinformatics* **10**, 1-9 (2009).
33. B. Yuen, J. Polzin, J. M. Petersen, Organ transcriptomes of the lucinid clam *Loripes orbiculatus* (Poli, 1791) provide insights into their specialised roles in the biology of a chemosymbiotic bivalve. *BMC Genomics* **20**, 820 (2019).
34. Y. Liao, G. K. Smyth, W. Shi, featureCounts: an efficient general purpose program for assigning sequence reads to genomic features. *Bioinformatics* **30**, 923-930 (2014).
35. X. Didelot, D. J. Wilson, ClonalFrameML: efficient inference of recombination in whole bacterial genomes. *PLoS Comput. Biol.* **11**, e1004041 (2015).
36. A. E. Darling, B. Mau, N. T. Perna, progressiveMauve: multiple genome alignment with gene gain, loss and rearrangement. *PLoS One* **5**, e11147 (2010).
37. A. Darling *et al*., Mauve: Multiple alignment of conserved genomic sequences with rearrangements. *Genome Res*. **14**, 1394-1403 (2015).
38. R. C. Edgar, MUSCLE: a multiple sequence alignment method with reduced time and space complexity. *BMC Bioinformatics* **5**, 113 (2004).
39. S. Capella-Gutiérrez, J. M. Silla-Martínez, T. Gabaldón, trimAl: a tool for automated alignment trimming in large-scale phylogenetic analyses. *Bioinformatics* **25**, 1972-1973 (2009).
40. A. Stamatakis, RAxML version 8: a tool for phylogenetic analysis and post-analysis of large phylogenies. *Bioinformatics* **30**, 1312-1313 (2014).
41. L. G. E. Wilkins, C. L. Ettinger, G. Jospin, J. A. Eisen, Metagenome-assembled genomes provide new insight into the microbial diversity of two thermal pools in Kamchatka, Russia. *Sci. Rep.* **9**, 3059 (2019).
42. L. G. E. Wilkins, *Ca*. T. endolucinida core gene nucleotide alignment to infer recombination to mutation ratio. FigShare. http://dx.doi.org/10.6084/m9.figshare.13296200.v1. Deposited 2020/11/27.
43. L. G. E. Wilkins, *Ca*. T. weberae and *Ca*. T. lotti core gene nucleotide alignment to infer recombination to mutation ratio. FigShare. http://dx.doi.org/10.6084/m9.figshare.13296401.v1. Deposited 2020/11/27.
44. L. G. E. Wilkins, *Ca*. T. taylori core gene nucleotide alignment to infer recombination to mutation ratio. FigShare. http://dx.doi.org/10.6084/m9.figshare.13296065.v1. Deposited 2020/11/27.
45. N. J. Croucher *et al.*, Rapid phylogenetic analysis of large samples of recombinant bacterial whole genome sequences using Gubbins. *Nucleic Acids Res.* **43**, e15 (2015).

46. L. G. E. Wilkins, J. T. Osvatic, B. Yuen, Analysis scripts (Jupyter Notebook). Figshare. https://doi.org/10.6084/m9.figshare.13295912.v5. Deposited 2020/11/27.
47. J. F. Villarreal-Chiu, J. P. Quinn, J. W. McGrath, The genes and enzymes of phosphonate metabolism by bacteria, and their distribution in the marine environment. *Front. Microbiol.* **3**, 19 (2012).
